# Platelets in Sepsis: An Update on Experimental Models and Clinical Data

**DOI:** 10.3389/fimmu.2019.01687

**Published:** 2019-07-17

**Authors:** Alice Assinger, Waltraud C. Schrottmaier, Manuel Salzmann, Julie Rayes

**Affiliations:** ^1^Center for Physiology and Pharmacology, Medical University of Vienna, Vienna, Austria; ^2^Institute of Cardiovascular Science, College of Medical and Dental Sciences, University of Birmingham, Birmingham, United Kingdom

**Keywords:** sepsis, inflammation, platelets, thrombocytopenia, infection

## Abstract

Beyond their important role in hemostasis, platelets play a crucial role in inflammatory diseases. This becomes apparent during sepsis, where platelet count and activation correlate with disease outcome and survival. Sepsis is caused by a dysregulated host response to infection, leading to organ dysfunction, permanent disabilities, or death. During sepsis, tissue injury results from the concomitant uncontrolled activation of the complement, coagulation, and inflammatory systems as well as platelet dysfunction. The balance between the systemic inflammatory response syndrome (SIRS) and the compensatory anti-inflammatory response (CARS) regulates sepsis outcome. Persistent thrombocytopenia is considered as an independent risk factor of mortality in sepsis, although it is still unclear whether the drop in platelet count is the cause or the consequence of sepsis severity. The role of platelets in sepsis development and progression was addressed in different experimental *in vivo* models, particularly in mice, that represent various aspects of human sepsis. The immunomodulatory function of platelets depends on the experimental model, time, and type of infection. Understanding the molecular mechanism of platelet regulation in inflammation could bring us one step closer to understand this important aspect of primary hemostasis which drives thrombotic as well as bleeding complications in patients with sterile and infectious inflammation. In this review, we summarize the current understanding of the contribution of platelets to sepsis severity and outcome. We highlight the differences between platelet receptors in mice and humans and discuss the potential and limitations of animal models to study platelet-related functions in sepsis.

## Sepsis

Sepsis is a highly complex life-threatening syndrome of organ dysfunction caused by dysregulated inflammatory host response to an overwhelming systemic infection (1). Sepsis is typically manifested by an early dominant hyper-inflammatory phase, the systemic inflammatory response syndrome (SIRS), characterized by fever and hyper-metabolism, which can eventually lead to septic shock. This pro-inflammatory state is followed by or co-exists with a compensatory anti-inflammatory response (CARS) and immunosuppression, leading to secondary complications (2).

According to the Sepsis-3 guidelines, sepsis is diagnosed by the Sequential Organ Failure Assessment (SOFA) score, which assesses organ dysfunction and risk of mortality (1). Sepsis is the primary cause of death from infection and occurs in 6–30% of patients in intensive care units (ICUs) (3). In-hospital mortality of patients with sepsis exceeds 10%, but increases to over 40% in severe cases which deteriorate into septic shock (1).

While early and effective medical interventions have managed to lower mortality over the last three decades (4), sepsis incidence is actually rising which might reflect the aging population in developed countries and increases the total number of patients suffering from or dying of sepsis.

### Pathology of Sepsis

Normal host response to pathogen invasion involves a complex process to localize and confine microbes, while initiating repair processes of injured tissue. Microbial components can be recognized by germline-encoded pattern recognition receptors (PRR) on host immune cells, which bind pathogen-associated molecular patterns (PAMPs) of microorganisms or danger-associated molecular patterns (DAMPs) that are released from injured tissues during the inflammatory insult. PRRs can be further divided in toll-like receptors (TLRs), C-type lectin receptors (CLRs), nucleotide-binding oligomerization domain (NOD)-like receptors, and retinoic acid-inducible gene I (RIG-I)-like receptors (RLRs) (5).

Engagement of PRRs elicits various signaling cascades essential for the neutralization of pathogens. A central event in this process is the activation of cytosolic nuclear factor-κB (NF-κB). Activated NF-κB translocates from the cytoplasm to the nucleus, where it binds to transcription sites and induces activation of a plethora of genes involved in inflammatory host response, including pro-inflammatory cytokines, chemokines, adhesion molecules, and nitric oxide (NO) synthase (6).

As first line of defense to infection, the complement cascade, neutrophils and endothelial cells are activated, inducing the expression of adherence molecules on endothelial cells and promoting neutrophil and subsequent monocyte migration and extravasation to the site of inflammation. Monocytes differentiate into macrophages *in situ* and secrete a mixture of pro-inflammatory [e.g., tumor necrosis factor (TNF)-α and interleukin (IL)-1] and anti-inflammatory (e.g., IL-10) mediators. The ingestion of apoptotic neutrophils by inflammatory macrophages induces their switch to anti-inflammatory macrophages with repair properties (7).

These processes are responsible for the cardinal signs of local inflammation: warmth and erythema due to local vasodilation and hyperemia, protein-rich edema due to increased microvascular permeability and pain due to mediators released by innate immune cells.

A fine-tuned balance of pro-inflammatory and anti-inflammatory mediators regulates and restricts the inflammatory processes, the invading pathogens are cleared, homeostasis is restored and tissue repaired. The systemic dissemination of the immune response to uninfected remote tissue and failure to restore homeostasis, leads to a malignant intravascular inflammation called sepsis.

Sepsis is characterized by an aggravated, uncontrolled, and self-sustaining inflammation which spreads via the circulation. The exact mechanism by which an inflammation switches from being locally restricted to systemically spread is unclear and is likely to be multifactorial. Pathogens and their toxic products contribute to this process, as endotoxins are found in the blood of patients and associated with shock and multiple organ dysfunction. The most common causes of sepsis are infections of the respiratory system, followed by genitourinary and abdominal infections (8). While sepsis can be caused by infection with bacteria, virus and fungi, the most frequent pathogens in sepsis are gram-positive bacteria such as *Staphylococcus aureus* and *Streptococcus pneumoniae* (4, 9). However, almost half of all patients with sepsis have unknown infections and are pathogen culture negative. This form of sepsis is on the rise and associated with higher incidences of acute organ dysfunction and mortality (10).

### Systemic Effects of Sepsis

The systemic immune response results in widespread cellular injury, which precedes organ dysfunction. The precise mechanism of cellular injury is highly complex and still incompletely understood. The main mechanisms involved include: cytopathic injury, which is mediated by direct cell injury by pro-inflammatory mediators and/or other products of inflammation, tissue ischemia due to insufficient oxygen supply, and an altered rate of apoptosis.

Tissue ischemia is caused by endothelial and microcirculatory lesions. Circulating inflammatory mediators activate the endothelium and induce loosening of tight junctions between endothelial cells, thereby increasing vascular permeability and leakage. As a consequence, systemic endothelial activation leads to hypotension and edema formation and thus to inadequate tissue oxygenation (11). Moreover, erythrocytes lose their normal ability to deform within the systemic microcirculation during sepsis (12), which impedes microcirculatory blood flow and depresses tissue oxygen supply. Microcirculatory lesions further occur as a result of imbalances in the coagulation and fibrinolytic systems, both of which are activated during sepsis.

Pro-inflammatory cytokines during sepsis promote cytopathic injury and also delay apoptosis in activated macrophages and neutrophils, thereby prolonging or augmenting the inflammatory response and contributing to the development of multiple organ failure. During the later phases of sepsis endotoxin tolerance (13) and extensive lymphocyte and dendritic cell apoptosis alter the immune response efficacy, decreasing the clearance of invading microorganisms and increasing the susceptibility to secondary infections (14).

Patients that die of sepsis show numerous overlapping mechanisms of immunosuppression involving both innate and adaptive immunity. Immune cells from spleens or lungs of septic patients, harvested shortly after the patients died, display a significant decrease in the production of pro- and/or anti-inflammatory cytokines and upregulated expression of inhibitory receptors including PD1. Also, the immunosuppressive cell populations of regulatory T cells and myeloid-derived suppressor cells are increased and CD28 and HLA-DR-mediated activation pathways downregulated (15).

During sepsis many cellular functions are affected, including mitochondrial functions which results in altered metabolic substrate utilization, biogenesis and mitochondrial reactive oxygen species (ROS) production. Reduced autophagy of damaged mitochondria and autophagy exhaustion further enhances inflammatory dysregulation and tissue injury (16).

Sepsis further promotes a pro-coagulant and pro-thrombotic state of the host. Local and circulating endotoxins induce the expression of tissue factor (TF) on endothelial cells and monocytes, promoting intravascular fibrin deposition and vascular occlusion. Moreover, microbial pathogens trigger the release of neutrophil extracellular traps (NETs) from neutrophils which provide a negatively charged surface for the activation and assembly of coagulation factors (17). Activation of the coagulation cascade provides a positive feedback loop inducing platelet activation via generation of thrombin, thereby promoting microthrombosis in response to inflammation and infection. This so-called immunothrombosis is part of the anti-microbial host response and aims to entrap invading pathogens and prevent their spreading although this mechanism is likely pathogen- and organ-dependent (18, 19). Further, the activated endothelium increased the recruitment and adhesion of circulating platelets, thus contributing to the formation of microthrombi throughout the body (20). During sepsis platelets can be sequestrated in the capillary-rich microvasculature of the spleen and liver. However, the majority of platelets accumulate in the lung microvasculature (21–23).

Thus, excessive responses of the immune system during sepsis are often associated with exaggerated and dysregulated activation of coagulation and thrombosis, manifesting itself as disseminated intravascular coagulation (DIC). During DIC microthrombi readily form within small and medium vessels, leading to disturbed tissue oxygenation, multi-organ, and eventually circulatory failure (24).

Activated platelets promote the development and progression of sepsis via their involvement in inflammation and thrombosis. Sepsis is characteristically accompanied by a drop in platelet count, reflecting their sequestration and their consumption in microthrombi although many other mechanisms contribute to the severity and persistence of thrombocytopenia (see below) (20). Severe thrombocytopenia is associated with a dysregulated host response leading to an increase in cytokine levels and endothelial dysfunction (25, 26). Hence, sepsis is associated with increased systemic thrombosis and coagulation as well as with elevated risk of hemorrhage due to the consumption of coagulation factors and platelets (24). Thrombocytopenia was found to correlate with sepsis disease severity and is associated with increased mortality risk (27, 28).

### Organ Specific Effects of Sepsis

Overwhelming systemic inflammation results in organ dysfunction. While no organ is protected from the consequences of sepsis, the most common complications are outlined below.

#### Circulation

During sepsis vasoactive mediators, including prostacyclin and NO, are released by endothelial cells. NO synthase can be induced by endotoxin and NO plays a central role in the vasodilation accompanying septic shock (29). Systemic NO release results in persistent vasodilation and diminishes the response to vasoconstrictors during sepsis (29). Furthermore, compensatory secretion of antidiuretic vasopressin is diminished during sepsis.

In the central circulation, decreased systolic and diastolic pressure diminishes the cardiac output. Low blood pressure also leads to disturbed blood flow in small vessels and restricts capillary functions in the microcirculation, leading to inefficient oxygen extraction due to edema, endothelial swelling and plugging by leukocytes.

Sepsis leads to endothelial dysfunction via direct and indirect interactions with pathogens. Degradation of the endothelial glycocalyx is associated with the upregulation of adhesion molecules (30), promoting the adherence of blood cells, which fosters mutual activation and further promotes inflammation and edema formation.

#### Gastrointestinal Tract

Hemodynamic abnormalities during sepsis depress the normal barrier function of the gut, allowing translocation of microbiota into the systemic circulation, further contributing to the progression of sepsis (31).

#### Lung

During sepsis microbial burden and/or increased inflammation results in endothelial injury of the pulmonary vasculature, leading to edema, increased leukocyte influx, and diminished oxygen supply. Excessive infiltration of innate leukocytes amplifies these processes by boosting inflammatory responses, causing injury to the lung tissue and hemorrhage, and thus causes loss of lung function. This may lead to the acute respiratory distress syndrome characterized by loss of the alveolar-capillary barrier function and increased vascular permeability, lung injury, and pulmonary edema (32). Clinical presentation thus includes diffuse bilateral pulmonary infiltrations and acute and persistent arterial hypoxia.

#### Liver

The liver works as a lymphoid organ in response to sepsis by mediating immune responses, leading to clearing of bacteria and toxins but also causing inflammation, immunosuppression, and organ damage. Attenuating liver injury and restoring liver function lowers morbidity and mortality rates in patients with sepsis (33).

#### Kidney

Acute kidney injury is a common phenomenon in sepsis and occurs in 40–50% of septic patients and increases mortality by 6–8-fold (34). The underlying mechanism is still incompletely understood. While previous notions regarded organ dysfunction solely as side effect of hypoperfusion, recent studies challenge this view and emphasize a role of heterogeneous areas of colocalized sluggish peritubular blood flow and tubular epithelial cell oxidative stress. Microvascular dysfunction, inflammation, and the metabolic response to inflammatory injury are crucial for the pathophysiologic mechanisms of kidney damage (34).

#### Brain

Sepsis is often characterized by an acute brain dysfunction which is associated with increased morbidity and mortality. Its pathophysiology is highly complex and involves inflammatory and non-inflammatory processes. Pathophysiological mechanisms include excessive microglial activation, impaired cerebral perfusion, blood-brain-barrier dysfunction, and altered neurotransmission. Systemic insults, such as prolonged inflammation, severe hypoxemia, and persistent hyperglycemia, may also contribute to aggravate sepsis-induced brain dysfunction or injury (35).

## Platelets In Sepsis

Sepsis is characteristically accompanied by increased activation of platelets, small anucleate blood cells with pivotal functions in hemostasis. It is becoming increasingly apparent that platelets have also essential roles in immunity and modulate physiologic and pathologic responses to inflammation and infection.

Platelets are crucial regulators of leukocyte function and thus of inflammatory immune responses (36). They readily interact with innate immune cells and exert immunomodulatory effects directly via cell-cell contact or indirectly via the release of chemokines and cytokines (37). Platelets promote endothelial adhesion and extravasation of leukocytes at sites of inflammation while securing vascular integrity at the site of transmigration. They modulate cytotoxic neutrophil effector function and induce a pro-inflammatory phenotype of neutrophils by modulating their activation, phagocytosis, oxidative burst, and formation of NETs. Platelets are also involved in monocyte differentiation into macrophages and modulate their effector functions. Thereby platelets also contribute to excessive inflammatory host response during sepsis and promote the development and progression of sepsis via their involvement in both inflammation and thrombosis. On the other hand, platelets can inhibit inflammation and promote tissue repair in a receptor- and organ-dependent manner. Therefore, the balance between the pro-inflammatory and anti-inflammatory roles of platelets regulates the outcome.

### Platelet Receptors in Sepsis

**Figure 1:**
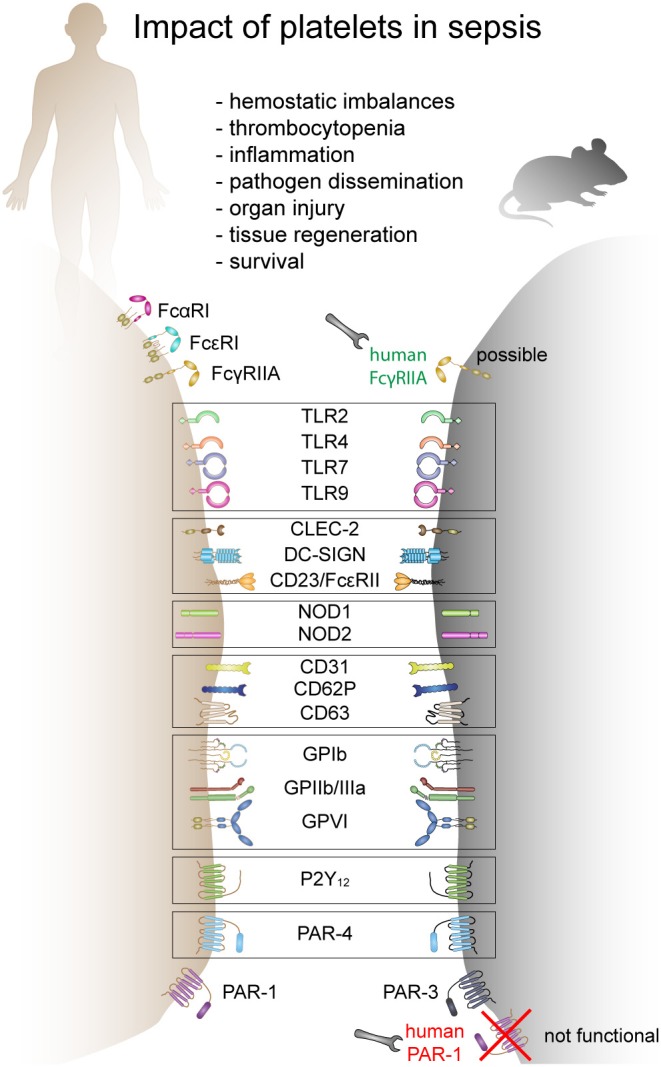
The role of platelets in sepsis and receptors involved in mice and humans. Human and mouse platelets express a variety of immune receptors involved in thrombosis or/and inflammation during sepsis. Many receptors are conserved between mice and human and other receptors are species-restricted. FcαR, Fc-alpha receptor; FcεR, Fc-epsilon receptor; FcγR, Fc-gamma receptor; TLR, toll-like receptor; CLEC-2, C-type lectin-like receptor-2; DC-SIGN, dendritic cell-specific intercellular adhesion molecule-3-grabbing non-integrin; NOD, nucleotide binding oligomerization domain containing 1; GP, glycoprotein; P2Y_12_, purinergic receptor P2Y_12_; PAR, proteinase-activated receptor.

Platelets express various receptors that are involved in the initiation and progression of sepsis. They include receptors for pathogen recognition, immune cell activation and platelet activation. While a plethora of receptors are ubiquitously expressed on platelets, others are only found on platelet subpopulations [e.g., TLRs (38)]. Circulating platelets differ in age, maturation state, or density. It is currently unknown if receptor composition differs due to platelet maturation, differences in thrombopoiesis and receptor distribution during platelet formation (39) or if a subset of megakaryocytes produces immune-regulatory platelets which express e.g., TLRs, while others produce platelets that only fulfill thrombotic functions. Moreover, there are gender-specific differences in expression levels of some receptors. Women, for example, express more copy numbers of TLRs compared to men (40) and receptor expression correlates with distinct cardiovascular risk and inflammatory biomarkers (40). Interspecies differences in receptor expression and copy numbers are even bigger. Mice and humans not only strongly differ in platelet count but also show differences in receptor density and co-receptor expression (41–43). Moreover, some receptors are not expressed in mice, while they fulfill important immunomodulatory functions in humans (e.g., FcγRIIA). This makes it often difficult to translate results from animal models to the clinical situation. A comparison of human and murine receptors relevant to sepsis pathology is given in Figure 1.

#### Toll-Like Receptors (TLRs)

TLRs are type I transmembrane proteins comprising of an ectodomain, which contains leucine-rich repeats that mediate the recognition of PAMPs, a transmembrane region, and a cytosolic Toll-IL-1 receptor (TIR) domain that activates downstream signaling pathways. TLRs are either expressed on the cell surface or associated with intracellular vesicles. Each TLR detects distinct PAMPs and therefore recognizes viruses, bacteria, mycobacteria, fungi, or parasites (44).

Human and murine platelets express functional TLRs. TLR2 regulates megakaryopoiesis (45), thromboinflammation (46), and bacterial phagocytosis (47). TLR7 stimulation triggers platelet degranulation and platelet-leukocyte aggregate formation and alters survival in virally infected mice (48). TLR9 regulates foreign DNA sequestration and CD62P surface expression in platelets (49). TLR4 is involved in rapid TNF-α induction (38), NET formation (50), and thrombocytopenia (38, 51). During gram-negative infection but not gram-positive infection, TLR4 activation induces the expression of neuraminidase, promoting alkaline phosphatase clearance and increasing LPS phosphorylation and toxicity (52–54). Interestingly, injection of neuraminidase post *Streptococcus pneumoniae* infection promotes survival and decreases fibrin clot frequency as well as liver and spleen injury in septic mice. Neuraminidase further induces moderate thrombocytopenia with platelet counts dropping by 70%, indicating that moderate thrombocytopenia might be beneficial in sepsis (55).

While TLR2 is only expressed on a small subset of platelets (10–20%), TLR4 is found on approximately 60% of human and 40% of murine platelets (23, 38). TLR9 is found on approximately 40% of resting human platelets and can increase to up to 60% in activated platelets indicating an intracellular storage pool of TLR9 (38, 49, 56). In mice, 60% of platelets are TLR9 positive and no further upregulation could be observed upon activation (38).

#### C-Tlatelet Antibodype Lectin Receptors (CLRs)

CLRs comprise a large family of receptors that bind to conserved bacterial structures via carbohydrate-recognition domains (CRDs) in a calcium-dependent manner. Based on their molecular structure, two groups of membrane-bound CLRs can be distinguished. Type I CLRs carry multiple CRDs or CRD-like domains, while Type II CLRs contain only a single CRD. CLRs are expressed on several immune cells. On platelets CD23 (FcεRII) and dendritic cell-specific ICAM-grabbing non-integrin (DC-SIGN) are expressed and functional (57–60). DC-SIGN is involved in lentiviral internalization by platelets (57).

#### Nucleotide-Binding Oligomerization Domain-Like Receptors (NOD-Like Receptors)

NOD-like receptors are cytoplasmic receptors with a strong structural similarity. All NOD-like receptors contain a central nucleotide-binding oligomerization domain (NOD), a C-terminal leucine-rich repeat domain and a variable N-terminal protein-protein interaction domain which interacts with downstream effectors. NOD1 and NOD2 are the two important NOD-like receptors fitting the typical structure with NOD1 containing one caspase recruitment domain (CARD), whereas NOD2 contains two CARD domains (61). While NOD1 recognizes d-glutamyl-meso-diaminopimelic acid primarily from gram-negative bacteria, NOD2 detects the muramyl dipeptide (MDP) motif in peptidoglycan from all bacteria. NOD1 is broadly distributed, whereas NOD2 is mainly expressed in innate immune cells (61) and platelets (62). In platelets NOD2 contributes to platelet activation and is possibly involved in arterial thrombosis during infection (62).

#### Glycoprotein Ib (GPIb)

GPIb is exclusively expressed on platelets and megakaryocytes and plays a crucial role in platelet adhesion under high shear. GPIb is part of the GPIb-V-IX complex, which binds von Willebrand factor (vWF), allowing platelet adhesion and platelet plug formation at sites of vascular injury. Many bacteria such as *Streptococcus sanguis* contain serine-rich protein A (SrpA) which is also recognized by GPIb and allows platelet-bacteria binding in a sialic acid-dependent manner (63). *Staphylococcus aureus* protein A (Spa) also binds vWF, which mediates indirect interaction with platelets via GPIb (64).

#### Integrin α_IIb_β_3_ (GPIIb/IIIa)

Glycoprotein IIb/IIIa (GPIIb/IIIa) represents the most abundant platelet glycoprotein and is central for platelet aggregation via fibrinogen bridging. GPIIb/IIIa becomes activated via inside-out signaling, leading to a conformational change that uncovers an arginine-glycine-aspartic acid (RGD) sequence, allowing the binding of fibrinogen, vWF, fibronectin and vitronectin, and platelet aggregation (65). Several bacterial proteins are able to bind to the RGD sequence on GPIIb/IIIa, including SdrG (Fbe) from *Staphylococcus epidermis* causing platelet aggregation (66). *Borrelia burgdorferi* also binds human platelets via GPIIb/IIIa (67). Clumping factors (Clf) on *Staphylococcus aureus* bind fibrinogen causing platelet aggregation (68).

#### Glycoprotein VI (GPVI)

GPVI is an immunoreceptor tyrosine-based activation motif (ITAM) receptor that plays a crucial role in the collagen-induced activation and aggregation of platelets. By binding to exposed subendothelial collagen, GPVI mediates the sealing of vascular injuries and ensures integrity of the circulatory system.

#### Protease Activating Receptors (PARs)

PARs are a subfamily of related G protein-coupled receptors highly expressed on platelets and are activated by cleavage of part of their extracellular domain by thrombin. Human platelets express PAR-1 and PAR-4 with PAR-1 mediating strong thrombin-induced activation. In contrast, murine platelets express PAR-3 and PAR-4 (69) with PAR-4 being the most potent receptor for thrombin.

#### Fc Receptors

Human platelets are reported to express various Fc receptors including FcαRI (CD89) (70), FcεRI (71), FcεRII (CD23) (59), and FcγRIIA (CD32a) (72). In infectious settings, FcγRIIA mediates immune complex-induced platelet activation or killing of opsonized bacteria (73, 74).

#### P2Y Receptors

P2Y receptors are a family of purinergic G protein-coupled receptors, stimulated by nucleotides such as adenosine diphosphate (ADP). The ADP receptors, P2Y_1_ and P2Y_12_, are differentially involved in pro-thrombotic platelet activation as well as expression of platelet P-selectin in response to various agonists (75). P2Y_12_ inhibitors are routinely used in the clinics for prevention of thrombotic complications.

#### P-Selectin (CD62P)

In resting platelets, CD62P is stored in the α-granule membrane and becomes exposed on the platelet surface upon activation. CD62P functions as adhesion molecule and is responsible for platelet interaction with activated endothelial cells and leukocytes via binding to its ligand P-selectin glycoprotein ligand 1 (PSGL-1), which enables platelets to fine-tune their cellular functions.

#### CD40 Ligand (CD40L)

Following activation platelets upregulate CD40L, promoting their interaction with innate immune cells, in particular monocytes. CD40L can be shed by metalloproteinase 9 (MMP9) which is upregulated during sepsis, resulting in a significant increase in soluble CD40L.

### Platelet Activation in Septic Patients

Increased platelet activation is observed in septic patients and is further potentiated in septic shock. Platelet activation is associated with the upregulation of surface expression of CD62P, CD63, CD31, increased fibrinogen binding and soluble GPVI (76–79), particularly in patients with DIC (80). Moreover, an increase in thrombospondin expression on circulating platelets is observed in patients with multiple organ failure (77). Platelets from septic patients show spontaneous aggregation but the *ex vivo* response to platelet agonists is severely reduced (81–83). Interestingly, the impairment in platelet aggregation is not associated with DIC (82). Whereas, platelet activation in sepsis is not questioned, platelet aggregation data is more contradictory due to the low platelet count in these patients which is not taken into account in many studies (80). Platelet activation is also associated with increased platelet-neutrophil and platelet-monocyte aggregates in septic patients (84, 85) further potentiating the inflammatory response. Together these results suggest that septic patients' platelets circulate in an activated state, increasing their thrombotic potential (84).

Different mechanisms may contribute to direct and indirect platelet activation during sepsis, including platelet activation by the pathogen (86–90), pathogen- and inflammation-driven activation of the endothelium and leukocytes and complement activation-mediated platelet activation (91). The complexity of platelet activation in sepsis suggests the contribution of multiple receptors, making it likely that combined therapy might be required to inhibit platelet activation in sepsis. However, increased bleeding risk in these patients adds another layer of complexity for targeting platelets in septic patients.

### Platelet Count in Septic Patients

The correlation between platelet count and sepsis severity and outcome has drawn increasing attention to the contribution of platelets to the pathophysiology of sepsis (92, 93). The critical role of platelets in sepsis is emphasized by the fact that platelet count is included in the SOFA score and is inversely associated with sepsis severity (94). Thrombocytopenia is often used to stratify patients with sepsis and septic shock based on the nadir of platelet count: mild thrombocytopenia (platelet count <100–150 ×10^9^/L), moderate thrombocytopenia (platelet count between 50 and 100 ×10^9^/L) and severe thrombocytopenia (platelet count <50 ×10^9^/L), with severe thrombocytopenia being associated with worse outcomes (25). Another important parameter is relative thrombocytopenia, that represents a drop of the initial platelet count over 4 days (95). Indeed, the kinetics of platelets in sepsis is often biphasic, characterized by an initial drop within the first few days (day 1–4) followed by an increase in platelets and thrombocytosis (96). Lack of this biphasic response leads to persistent thrombocytopenia and is associated with poor prognosis and increased 28-day mortality (96, 97).

Thrombocytopenia may occur before admission at the hospital or during the ICU hospitalization (96, 98, 99). Of note, a single measurement of platelet count is not predictive of mortality (92, 96, 100). The initial platelet drop does not discriminate between survivors and non-survivors while late thrombocytopenia is more predictive for mortality. Indeed, stratification of septic patients revealed that not only the severity of thrombocytopenia but more importantly persistence of thrombocytopenia is associated with worse outcome. Severe thrombocytopenia is independently associated with disease severity and mortality at the ICU admission and is associated with a dysregulated host response (25).

In general, 20–58% of septic patients develop thrombocytopenia, of which 10% develop severe thrombocytopenia (28, 93, 96, 98, 101). The discrepancy in the reported values might arise from patient heterogeneity, different inclusion criteria, pathogens and other factors. Moreover, a decrease in immature platelets, indicating impeded thrombopoiesis, is associated with severe thrombocytopenia and 28-day mortality (102). One recent study showed that immature platelet fractions could predict sepsis occurrence in critically ill subjects (103). Interestingly, phosphatidylserine-expressing platelet microvesicles are decreased in non-survivors and correlate with thrombocytopenia but are not associated with DIC (104). Therefore, severity and persistence of thrombocytopenia as well as immature platelet fractions and platelet microvesicle composition are strong predictors of mortality in sepsis.

### Causes of Thrombocytopenia in Septic Patients

The association between thrombocytopenia and clinical outcome does not reveal any causality as many parameters could be both the cause and/or the consequence of dropping platelet counts.

**Figure 2:**
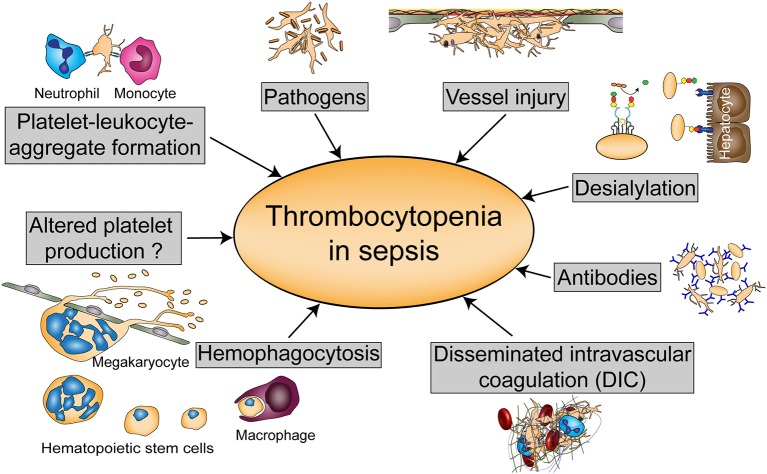
Possible causes of thrombocytopenia in sepsis. Thrombocytopenia in sepsis might be either regulated by altered platelet production or hemophagocytosis, or by platelet scavenging in the circulation either due to platelet-leukocyte or platelet- pathogen interactions, vessel injury, or desialylation. Platelets can also be targeted by antibodies during sepsis or reduced due to disseminated intravascular coagulation (DIC).

Thrombocytopenia can either occur due to diminished platelet production or increased platelet turnover. Platelet activation diminishes platelet life span as activated platelets are rapidly cleared from the circulation. Animal models revealed that thrombocytopenia in sepsis is largely TLR4-dependent, which suggests that immune-mediated platelet activation represents a main cause for the drop in platelet count (38). Many mechanisms have been proposed for thrombocytopenia in sepsis although a combination of multiple mechanisms might occur in severely thrombocytopenic patients. An overview of these processes is given in Figure 2.

#### Platelet-Leukocyte Aggregation

The formation of platelet-leucocyte aggregates (PLA) in the blood depends on platelet activation and is an early phenomenon occurring in sepsis. Circulating PLA are increased in sepsis patients at an early phase, but significantly decrease in non-survivors and patients developing multiple organ failure, likely due to enhanced peripheral sequestration or sepsis-associated thrombocytopenia (84). Platelet-neutrophil aggregates can also potentiate thrombocytopenia through the release of platelet-activating NETs (105).

#### Pathogen-Induced Thrombocytopenia

Fungi and bacteria can interact with platelets and induce platelet activation and aggregation (86, 87, 89). Although many bacteria activate platelets in a GPIIb/IIIa- or FcγRIIA-dependent manner and involve plasma proteins such as IgG, complement proteins and fibrinogen, other bacteria directly bind and activate platelet receptors such as GPVI and TLRs, increasing platelet activation and PLA formation (46, 87, 106). Mechanisms of pathogen clearance by platelets may occur indirectly through the release of various antimicrobial peptides and platelet-derived mediators regulating the activation of the endothelium and immune cells. Some pathogenic bacteria, particularly blood stream infections, may also trigger apoptosis in platelets resulting in thrombocytopenia (107, 108). Thrombocytopenia occurs in 20–30% of patients infected with *Staphylococcus aureus, Escherichia coli*, or *Streptococcus pneumonia*, suggesting that platelet activation by pathogens contributes to thrombocytopenia but does not represent a major mechanism (109). TLR4-mediated responses reduce platelet counts in murine endotoxemia (38, 110), but the role of TLR4 in patients has never been investigated.

#### Tissue Injury-Mediated Platelet Activation

Infections are commonly associated with tissue injury and cell destruction that fuel inflammation. DAMPS and other mediators released from activated and injured cells such as histones and high mobility group protein B1 (HMGB1) can activate platelets and enhance agonist-induced platelet activation and granule secretion, potentiating thrombocytopenia and delaying its resolution (111–113).

#### Clearance Through Desialylation

Platelet desialylation is increased in septic patients with thrombocytopenia compared to patients without thrombocytopenia (114). Desialylation occurs during pneumococcal infections, leading to the release of neuraminidase and the exposure of galactose residues, increasing the clearance of platelets by the Ashwell Morrell receptors (AMR) on hepatocytes. GPIbα is the main receptor involved in platelet clearance and is desialylated by neuraminidase, although other glycoproteins are also susceptible to desialylation. Moreover, platelet desialylation increases platelet reactivity, thereby potentiating thrombocytopenia (115).

#### Anti-platelet Antibodies

Another possible mechanism that contributes to thrombocytopenia is the immune clearance and destruction of platelets. Anti-platelet antibodies (e.g., anti-PF4/heparin) are detected in patients with bacterial septicemia and their level increases in thrombocytopenic patients with no significant difference between gram-positive and gram-negative infection (116–118). Moreover, IgG-opsonization enhances the clearance of LPS-binding platelets in an Fc-dependent manner and further potentiates platelet clearance in gram-negative infection (51). Inflammation fosters immune thrombocytopenia as C-reactive protein, produced during the acute phase of inflammation, enhances antibody-mediated platelet clearance by FcγR-dependent phagocytosis (108). More recently a novel mechanism of platelet sequestration through FcγRIIA activation by immune complexes was suggested (119). These studies consolidate that different mechanisms contribute to thrombocytopenia during different infections.

#### Disseminated Intravascular Coagulation (DIC)

DIC is defined as an excessive fibrin deposition leading to the occlusion of blood vessels and organ damage that is associated with consumption of coagulation factors and platelets. DIC is often associated with low platelet count, abnormal coagulation and fragmented cells; however thrombocytopenia is not always associated with DIC (100). DIC was one of the first mechanisms suggested to explain thrombocytopenia in septic patients but the low association between thrombocytopenia and DIC (15–30% of patients with thrombocytopenia present with DIC) suggests that DIC might contribute to thrombocytopenia only in some cases of severely ill patients (96, 120). DIC is mostly observed in patients with septic shock, however the association between DIC and mortality largely depends on the study and the inclusion criteria (96). More recently, it was shown that although DIC occurs in septic patients and mice, the time course and the composition of thrombi differ between organs even within the same infection (18, 121). This might be due to the different susceptibility of the endothelium, the local and systemic generation of coagulation factors, fibrinolytic factors and extracellular matrix proteins as well as due to the activation of immune cells.

#### Platelet Production (Thrombopoiesis)

The presence of normal megakaryocyte counts in the bone marrow of septic patients with low platelet count suggests that thrombopoiesis remains unaffected (95). Moreover, the increase in immature platelet fraction, the absolute immature platelet count and the increase in thrombopoietin (TPO) levels consolidate this hypothesis (122, 123). Increased TPO levels might result from reduced platelet count or enhanced TPO production in the liver by inflammatory mediators. However, in some severely ill patients with advanced thrombocytopenia, a defect in thrombopoiesis might occur, which could explain why these patients do not recover to a normal platelet count (102).

#### Cytokine-Driven Hemophagocytosis

An increase in the proliferation and activation of monocytes and macrophages in the bone marrow was observed in septic patients with thrombocytopenia. The uncontrolled proliferation is associated with an increase in macrophage-colony stimulating factor (M-CSF) which accelerates the ingestion of hematopoietic cells by macrophages and may contribute to thrombocytopenia (124).

Taken together, mild and moderate thrombocytopenia might result from one or the combination of different mechanisms, whereas severe and non-resolved thrombocytopenia involves concomitant mechanisms of platelet activation, sequestration and destruction.

### Anti-platelet Drugs in Sepsis

Several observational and retrospective clinical studies have shown that anti-platelet agents such as aspirin (COX-1 inhibitor), platelet P2Y_12_ receptor antagonists like clopidogrel or GPIIb/IIIa antagonists reduce mortality or complications in critically ill patients (99, 125, 126). In human experimental endotoxemia, P2Y_12_ inhibitors reduce the pro-inflammatory and pro-thrombotic mechanisms (127). Further, in septic patients low dose aspirin is associated with a decrease in mortality during hospitalization (128). Patients on low dose aspirin have shorter in-hospital stays and reduced need for intensive care treatment. Administration of aspirin for 24 h at the time of SIRS recognition is associated with increased survival in a large cohort of over 5,000 septic patients (129).

Inhibition of platelet function in sepsis represents an attractive target due to their role in thrombosis and inflammation. However, as platelet receptors play different roles in thrombosis, inflammatory hemostasis and inflammation, precautions have to be taken when targeting platelets in infection (130). Large randomized controlled clinical trials with anti-platelet therapy in stratified patients are warranted to determine a conclusive beneficial effect of anti-platelet drugs in sepsis.

## Rodent Models To Study The Role Of Platelets In Sepsis

In septic patients, disease progression before hospital admission often remains unknown. Therefore, it can be challenging to determine cause and effect of the clinical symptoms. Animal models help to unravel these early processes and the availability of genetically modified mouse strains contributed to the identification of distinct signaling pathways or genes as potential biomarkers or drug targets. Thus, rodent and especially mouse models have proven to be convenient and widely-used tools to study cellular and molecular mechanisms of sepsis in defined settings.

**Figure 3:**
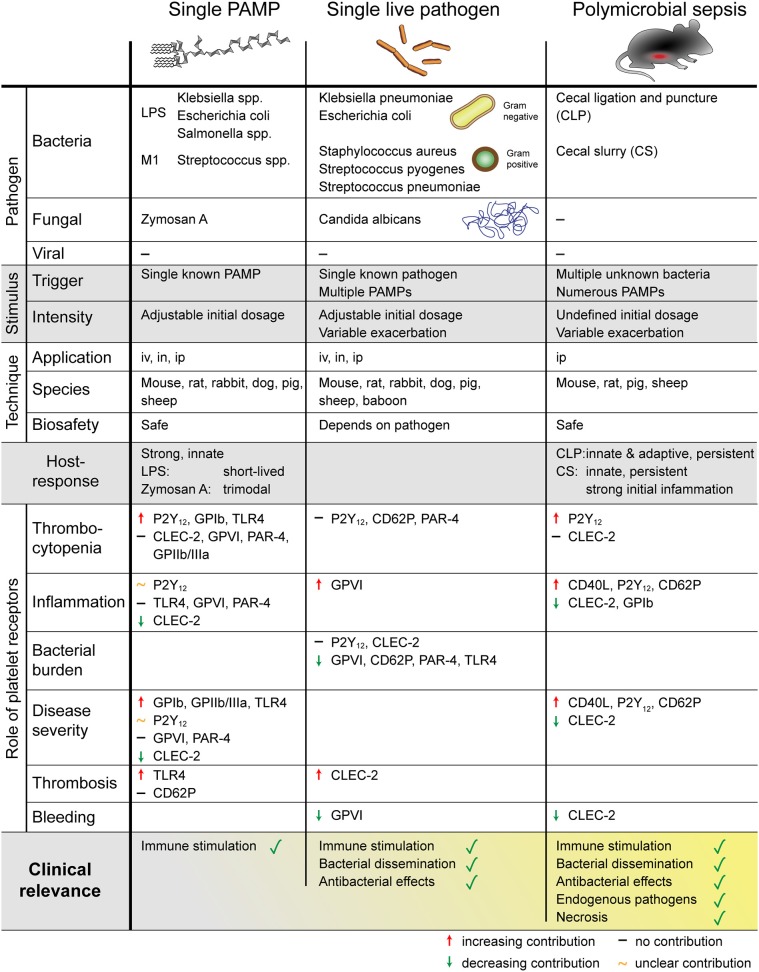
Overview of different mouse models to study sepsis. The role of platelets was addressed using single PAMP injection, single live pathogen or polymicrobial pathogens. Platelets regulate thrombosis, inflammation, bleeding, and sepsis outcome in a receptor- and pathogen-dependent manner. PAMP, pathogen-associated molecular pattern; LPS, lipopolysaccharides; M1, streptococcal M1 protein; iv, intravenous; in, intranasal; ip, intraperitoneal; P2Y_12_, purinergic receptor P2Y_12_; GP, glycoprotein; TLR, toll-like receptor; CLEC-2, C-type lectin-like receptor-2; PAR, proteinase-activated receptor.

A number of different sepsis models have been developed that vary in technical complexity, controllability, and representativeness for the human sepsis patient setting. As sepsis commonly originates from infections of lung and genitourinary tract and to a less extend the abdomen (8), intraperitoneal or pulmonary routes are favored for primary induction and experimentally easy accessible. An overview of the different animal models used to investigate the role of platelets and their receptors in sepsis is given in Figure 3.

### Different Models to Study Platelet Function in Sepsis

#### Single PAMP

The most controllable *in vivo* model is the injection of a single bacterial or fungal PAMP via intravenous, intraperitoneal, or intranasal/intratracheal application. Most commonly LPS from gram-negative *Escherichia coli* or *Klebsiella* is used, which stimulates TLR4 on host cells including platelets (23). LPS represents a weak platelet agonist and primes platelets for stimulation with other agonists (131), though its effect on platelet degranulation may not be detectable in every experimental setting (23). LPS usually signals via MyD88, however lack of platelet MyD88 did not alter host responses to LPS including thrombocytopenia and immune cell recruitment (132). Intravenous challenge with low-dose (0.125–0.25 mg/kg) LPS leads to rapid but transient thrombocytopenia accompanied by platelet sequestration in capillary-rich organs such as lungs and liver (133). For intraperitoneal application doses commonly range between 1 and 10 mg/kg which induces prolonged thrombocytopenia, platelet sequestration and innate immune cell infiltration into lungs and liver, changes in the coagulation state and an inflammatory cytokine response involving TNF-α and IL-6, which may eventually lead to death (23, 134). As such, LPS triggers a strong but short-lived response favoring the innate immune system.

However, the intensity of platelet responses depends on the specific LPS serotype of the O antigen, the outermost polysaccharide domain of the molecule. LPS serotypes O8 and O9 readily induce platelet activation, thrombocytopenia, platelet sequestration in the lungs and liver as well as increased mortality, whereas O111 and LPS of the strain K-12, lacking an O antigen, trigger weaker responses (135). Additionally, LPS can be classified in smooth (full-length O chains) or rough (reduced or absent O chains). A comparison of eight different smooth and rough LPS serotypes from *Escherichia coli, Klebsiella, Salmonella minnesota*, and *Salmonella typhimurium* in mice identified LPS of *Klebsiella* O3 as most potent to induce a platelet response and shock, triggering a complement-dependent accumulation and degradation of platelets in lungs and liver (136).

In addition to LPS administration, cell-surface proteins from gram-positive bacteria such as the M1 protein of *Streptococcus pyogenes* can be used to mimic sepsis that does not engage TLR4, but rather stimulates the immune system via super-antigens or peptidoglycans (137). However, M1-induced sepsis represents an unfavorable model to study platelets in sepsis as M1 challenge leads to neutrophil-dependent organ damage independently of platelets (138).

Fungal sepsis can be investigated by injection of the yeast-derived cell surface glucan zymosan A (e.g., from *Saccharomyces cerevisiae*) which activates TLR2 and the alternative complement pathway. Unlike LPS, zymosan A induces a triphasic immune response which resembles the prolonged sepsis in humans. The first phase is characterized by a strong pro-inflammatory response with high levels of TNF-α and IL-6, followed by chronic low-grade inflammation which eventually culminates in organ damage and death (137, 139).

Nevertheless, screening of literature revealed that LPS from *Escherichia coli* O111:B8 appears to be the most commonly used agent for murine endotoxemia.

#### Single Live Pathogen

Injection of a single, live pathogen is more representative of infections in patients that originate from one infectious agent. While, by nature of live pathogens and their proliferation *in vivo*, this model has a lower level of controllability than injection of isolated PAMPs, the possibility of challenging animals with individual pathogens of choice provides the huge advantage of studying mechanisms underlying specific pathogens or bacterial strains.

Commonly used bacteria to study the role of platelets in murine sepsis models include *Escherichia coli, Klebsiella pneumoniae, Staphylococcus aureus, Streptococcus pyogenes*, and *Streptococcus pneumoniae* (140–144). Although intravenous injection of bacteria leads to strong bacteremia, complement-mediated host responses may prevent efficient colonization of organs and as such this model fails to accurately reproduce human sepsis (145). Additionally, due to the immediate effect on endothelial cells and the vasculature, intravenous administration triggers a potent, rapid, pro-inflammatory immune response that may be stronger than the host response induced by a local infection.

Nonetheless, challenge with single live pathogens represents a good model to study e.g., pneumosepsis, in which infections originate in the lungs before spreading systemically. Accordingly, intranasal infection with 10^4^-10^6^ colony forming units (CFU) of *Klebsiella pneumoniae* or *Streptococcus pneumoniae*, the most common gram-negative and gram-positive causative pathogens of community-acquired pneumonia, respectively (146), induces local pulmonary inflammation with accompanied cytokine response and infiltration of neutrophils and macrophages into the inflamed lungs. As local immunity becomes unable to contain the infection, bacteria disseminate into the bloodstream and can be detected in distant organs such as spleen, kidneys, and liver (141, 144).

Therefore, infection with live bacteria also allows studying of antibacterial host responses including phagocytosis, formation of NETs and release of antimicrobial agents, which play important roles in human sepsis.

In addition to live bacteria, experimental sepsis can also be induced by the human commensal fungus *Candida albicans* which may cause sepsis in humans upon breaching mucosal barriers of the gut e.g., following surgery or trauma (147). Innate immune recognition of *Candida albicans* involves various CLRs and TLRs, several of which are expressed on platelets (148). Infection of susceptible mice with live *Candida albicans* results in thrombocytopenia and decreased clotting times, indicating activation of primary and secondary hemostasis similar to bacterial sepsis (149).

#### Polymicrobial Infection

Models of polymicrobial infection most closely resemble human sepsis originating from an intestinal center of infection.

Cecal ligation and puncture (CLP) represents one of the most commonly used sepsis models as it most closely resembles sepsis in humans regarding biochemical, hemodynamic and immune responses, including hypotension, leukopenia, thrombocytopenia with a concomitant pro-thrombotic and pro-coagulatory phenotype, raised levels of pro-inflammatory cytokines as well as markers of organ dysfunction (150–152). Perforation of the cecum mimics a breach of intestinal barrier with subsequent dissemination of intestinal microbial flora into the peritoneum. In CLP this peritonitis is combined with necrosis of the ligated tissue and eventual dissemination into the periphery, resulting in septicemia and distant organ damage (152).

In addition to resembling the human sepsis situation, CLP has the advantage of being adjustable in severity based on the ratio of ligated tissue and the size and number of cecal perforations. However, CLP is prone to variation due to the operator and local immune responses may manage to contain bacteria in an abscess, thereby preventing the progression to septic shock (153). CLP-induced microvascular dysfunctions are not mediated by LPS (154), underlining the importance of gram-positive bacteria in this model. Compared to LPS challenge, the inflammatory cytokine response to CLP develops slower, even at similar mortality and morbidity (155), which may be due to the gradual disease progression.

Another model of polymicrobial sepsis is the cecal slurry (CS) method which comprises the intraperitoneal injection of a defined amount of donor feces and results in a stronger early inflammatory response than CLP (156). Curtailed variability in infectious dose and technical ease make CS a suitable model for polymicrobial infection in settings where surgery is problematic.

### Underlying Mechanisms Identified by Mouse Models

#### The Impact of Platelet Depletion on Sepsis

Platelet depletion has detrimental effects on survival in gram-negative (*Klebsiella pneumoniae*) and gram-positive (*Streptococcus pneumoniae*) pneumosepsis, accompanied by increased pulmonary hemorrhage and clinical pathology score (141, 144, 157). While pulmonary neutrophil infiltration appears largely platelet-independent in live bacterial models, platelets facilitate leukocyte recruitment in LPS/zymosan-induced lung injury (158). Accordingly, platelets seem to contribute to tissue injury in the absence of live pathogens. In contrast, low platelet counts are associated with increased secondary hemostasis, liver and kidney damage as well as exacerbated bacteremia and systemic bacterial dissemination in bacteria-induced sepsis (140, 141, 144, 158, 159). In line with these findings, thrombocytopenia also exacerbates the inflammatory response in sepsis, raising plasma levels of TNF-α, IL-6, IL-10, myeloperoxidase (MPO), monocyte chemotactic protein 1 (MCP-1), and interferon-γ (IFN-γ) (141, 144, 159), potentially as a consequence of more severe infection.

Nonetheless, the role of platelets in sepsis is multi-faceted and current research has only begun to untangle the complex interplay of inflammation, thrombosis, and coagulation that occurs during sepsis. Indeed, the role of platelets may depend on the specific pathologic setting and thus experimental model, as platelet depletion in *Streptococcus pyogenes* actually ameliorates weight loss, decreases bacterial burden and dampens the inflammatory host response (143). The reason for this discrepancy with other reports is currently unknown.

#### Platelets Receptors in Sepsis

Platelet receptors regulate platelet activation as well as subsequent platelet-mediated modulation of immune responses during sepsis.

Platelet TLR4 mediates microvascular thrombosis and thrombocytopenia in response to LPS, thereby fostering tissue injury caused by vessel occlusion. Furthermore, in *Escherichia coli* infection platelet TLR4 contributes to bacterial trapping by supporting NET formation (38, 50, 110).

Additionally, platelet activation during sepsis may be induced by activation of secondary hemostasis with generation of thrombin or release of vWF from activated endothelial cells, respectively. Inhibition of PARs does not alter mortality, inflammation, or thrombocytopenia in endotoxemic mice, suggesting that thrombin formation is not the main cause of thrombocytopenia in this model (160). Contrarily, in *Streptococcus pneumoniae*-induced pneumosepsis PAR-4 limits bacterial growth and lung damage (161).

Direct cellular interactions of activated platelets with leukocytes or endothelial cells via surface expressed CD62P or CD40L contribute to the inflammatory host response in sepsis, fostering both bacterial clearance and organ damage (162–164). Elevated CD62P promotes formation of platelet-neutrophil aggregates in the circulation of septic mice, assisting pulmonary neutrophil infiltration independent of local chemokines, and thereby limiting bacterial dissemination but also contributing to lung damage in pneumosepsis or CLP (162, 163, 165). Reduced surface CD40L curtails direct platelet-leukocyte interaction and dampens neutrophil infiltration and tissue damage in bacterial sepsis (164).

Following LPS challenge, thrombocytopenia, thrombosis and mortality are decreased in IL-4R/Ibα mice that lack the extracellular part of GPIbα (166), showing a detrimental role of GPIbα in endotoxemia. The role of GPIbα involves its interaction with vWF as disruption of this axis confers the same protective effect (166). While platelets commonly have pro-inflammatory effects on leukocytes, GPIb seems to confer anti-inflammatory leukocyte modulation as it supports platelet-leukocyte interaction, but dampens the inflammatory cyto-/chemokine response (167).

Furthermore, blockade of GPIIb/IIIa using integrilin also reduces mortality but did not alter thrombocytopenia in LPS-challenged mice (166), suggesting that thrombocytopenia is not the main cause of mortality in this model.

Recent studies have also identified glycoprotein GPVI and CLEC-2 as novel modulators of inflammatory responses in gram-negative sepsis or CLP. GPVI contributes to local immunity in pneumosepsis by enhancing platelet-neutrophil-aggregate formation and bacterial clearance (157). In contrast, CLEC-2 confers immune-inhibitory effects by dampening levels of pro-inflammatory cyto- and chemokines as well as limiting immune cell recruitment, inflammatory bleeding, and bacterial dissemination, thus ameliorating organ damage in endotoxemia and CLP (168). However, CLEC-2 also mediates inflammation-driven thrombosis in sepsis (169). Interestingly, while GPVI is not required for immune responses in endotoxemia, concomitant deletion of GPVI and CLEC-2 reverses the exaggerated inflammation and disease severity caused by lack of CLEC-2 alone. Thus, despite similar downstream signaling molecules, GPVI and CLEC-2 seem to play opposite roles during sepsis, most probably by regulating both the inflammatory response and thrombosis (168).

#### Platelet-Derived Soluble Mediators in Sepsis

In addition to direct cellular interactions, activated platelets secrete a plethora of soluble mediators from their granules that potentially modulate host responses to sepsis. Nbeal2-deficient mice, which lack α-granules, challenged with *Klebsiella pneumoniae*-induced pneumosepsis exhibit similar circulating platelet-leukocyte aggregates as wildtypes, but increased pulmonary leukocyte influx and elevated multi-organ damage. However, limiting Nbeal2-deficiency to the platelet compartment does not reproduce these results, suggesting that platelet granule content does not regulate host responses during *Klebsiella pneumoniae*-induced pneumosepsis (170).

Nonetheless, multiple studies focusing on specific mediators and using different sepsis models such as LPS-induced endotoxemia or CLP reported significant contributions of platelet granules proteins.

Platelet activation during sepsis triggers the release of RANTES (CCL5) and platelet factor 4 (PF4, CXCL4) and subsequent heteromer formation in the circulation. PF4 and RANTES in turn stimulate alveolar macrophages to produce the chemokines macrophage inhibitory protein-2 (MIP-2) and KC (CXCL1; homologous to human IL-8/CXCL8), thus promoting neutrophil recruitment but also edema formation (171–173). Moreover, PF4 accelerates generation of activated protein C, counteracting the increasing pro-coagulant state during sepsis which may promote DIC. Accordingly, PF4 has been found to increase survival in endotoxic shock (174).

In addition to cytokines and chemokines, serotonin released from dense granules upon platelet activation may play a role in sepsis. Using an FcγRIIA-humanized mouse model, it has recently been discovered that immune complexes cause platelets to transiently sequester to the lung, where they release serotonin before returning to the circulation (119). As serotonin activates endothelial cells, platelet-derived serotonin may subsequently promote the adhesion and extravasation of neutrophils (175). Additionally, platelet-derived HMGB1 has also recently been implicated in augmenting leukocyte recruitment and bacterial clearance in murine CLP (176).

The contribution of platelet release products to organ damage during sepsis may not be limited to support of neutrophil influx. During sepsis platelets carry intracellular granzyme B, probably due to transcriptional alterations in megakaryocytes, which causes local apoptosis at sites of platelet accumulation such as the lungs, spleen, and kidneys, contributing to multiple organ dysfunction and sepsis progression (177, 178).

These findings strongly underline the importance of platelet activation for essential host responses during bacterial sepsis such as immune cell recruitment, bacterial clearance, and organ dysfunction. Indeed, mice lacking PAR-4 show reduced levels of circulating PF4 48 h after infection with *Streptococcus pneumoniae*. In line with the protective effect of PF4 in endotoxic shock described above, these mice also suffer from increased bacteremia and bacterial burden in the lungs, as well as exacerbated pulmonary damage (161).

#### Pharmacological Platelet Inhibition During Sepsis

Given the availability of anti-platelet drugs, pharmacologic targeting of platelet function represents an attractive approach to mitigate platelet-assisted excessive inflammation that contributes to sepsis progression.

Interestingly, inhibition of cyclooxygenase-1 (COX-1) ameliorates thrombocytopenia and kidney dysfunction in endotoxemia (179), yet transfusion of COX-1-deficient platelets into platelet-depleted mice leads to worse survival than transfusion of wildtype platelets (140). The impact of P2Y_12_ on murine sepsis remains subject to discussion and depends on the specific model used. Mice deficient in P2Y_12_ show a protective role for P2Y_12_ in endotoxemia by ameliorating inflammation and lung injury, although the results are not mirrored by the use of a P2Y_12_ inhibitor (180). In contrast, blocking ADP feedback by P2Y_12_ receptor antagonists, clopidogrel, prasugrel, or ticagrelor, appears to be beneficial during sepsis, as it inhibits platelet activation and binding to circulating neutrophils and monocytes during pneumonia and CLP (181–183). This is accompanied by diminished TNF-α and IL-1β levels, as well as impeded neutrophil infiltration and platelet sequestration, ultimately reducing lung and kidney injury, whereas bacterial clearance does not seem to be affected by P2Y_12_ blockage (144, 181–184). Furthermore, clopidogrel failed to ameliorate thrombocytopenia in gram-negative pneumosepsis (185), whereas in a modified CS model, clopidogrel also improved sepsis-induced thrombocytopenia (186).

Thus, P2Y_12_ receptor antagonists show promising results in pre-clinical studies to ameliorate sepsis pathogenesis, while the potential of COX-1 inhibition remains unclear. Further, the effects of anti-platelet medication on established sepsis as well as the impact of P2Y_12_ blockers on bleeding risk have not been addressed in detail thus far. Therefore, large clinical trials are required to confirm if results from animal studies will be translatable to the human patient setting.

### Translational Limitations of Rodent Sepsis Models

Mouse and rat models have proven to be valuable tools to investigate cellular and molecular processes in sepsis. However, animal models have inherent limitations independent of the specific sepsis model and species that need to be taken into consideration when evaluating and interpreting results.

#### Age and Sex

Despite efforts to optimize, murine models do not appropriately represent the archetypal septic patient which is an elderly person with one or more co-morbidities. In contrast, mice are typically used at the age of 6–12 weeks with most studies focusing only on males. A literature screen for studies on platelets in sepsis revealed an average age of 9 weeks with 60% of studies being performed on males, 7% on females, 12% on both sexes, and no specified information was available in 21% of studies. Similarly, studies using rats as model organism also mostly use adolescent males. Therefore, most *in vivo* studies are more representative of healthy young men regarding age and sex rather than heterogeneous patient populations. Time and financial constraints are contributing to this bias as aging animals are a costly investment. Further, reproducibility is higher in young cohorts that vary less in weight and exposure to environmental stressors.

#### Species

While a variety of species are currently used as animal models in sepsis research, rodent models are the preferred approach for studying platelets. LPS-induced endotoxemia and CLP are widely used in both rats and mice. Rats are favorable models for *in vitro* analyses of platelets as their larger body weight and thus blood volume allows various concomitant measurements. Indeed, rat models have unveiled a number of crucial intracellular responses of platelets to sepsis, including activation of the NLRP3 inflammasome (187), upstream regulation of NADPH subunit p47^phox^-dependent ROS production (188) and the contribution of protein kinase C for platelet-mediated leukocyte infiltration and organ injury (189). Furthermore, rats are more commonly used when investigating effects of therapeutic intervention strategies on clinical parameters e.g., hemodynamics. However, genetic tools such as transgenic or knockout strains are rare in rats, but readily available for mice. Thus, despite limited sample material, murine models offer a wider range of experimental approaches which are invaluable for investigating underlying cell-specific molecular mechanisms. Of note, inflammatory responses appear to be stronger and peak earlier in mice than in rats (190). Therefore, interspecies differences have to be considered when interpreting findings of different animal models as well as their translation into the human setting.

#### Genetic Background

Another point to consider is the genetic background of mice as individual inbred strains vary in their immune competence due to polymorphisms and/or mutations, e.g., in TLRs or complement factors. While the most widely used strains C57BL/6 and BALB/c express functional TLR4 and are therefore sensitive to LPS, point mutations in the tlr4 gene have rendered some strains (e.g., C3H/HeJ, C57BL10/ScSr) resistant to LPS (191), making them useful tools to study endotoxin-independent host responses (154). However, C57BL/6 are more susceptible to fungal sepsis induced by zymosan or *Candida albicans* infection than outbred CF-1 mice, showing weaker Th1 response and poor survival (192). Further, mouse strains carrying loss-of-function mutations of complement factor C5 such as DBA/1 or DBA/2 display altered susceptibility to certain bacterial strains.

Genetic background not only impacts on innate but also on adaptive immunity. C57BL/6 mice tend to respond to pathogens with an enhanced Th1-type response, leading to increased phagocytic clearance of intracellular pathogens. In contrast, BALB/c mice are skewed toward Th2-type responses that support humoral immunity especially against extracellular parasites (191).

#### Timeline

With the exception of CLP, rodent sepsis models rarely mirror the timeline of sepsis pathogenesis in human patients. Injection of PAMPs or live bacteria does not represent the slow outgrowth and dissemination of bacteria from a center of infection, but rather a sudden, overwhelming infection that typically leads to death of the mice in a matter of hours or days. However, adjustment of the infectious dose may yield a transient, non-lethal infection that may be resolved within days. During this timeframe, immediate host responses involving platelets, coagulation and the innate immune system can be studied, as thrombocytopenia may occur within minutes after infection and induction of acute phase cytokines and neutrophil infiltration toward sites of acute inflammation can be observed within hours.

Additionally, experimental setups seldom reproduce the reality of sepsis in patients, where interventions have to be efficient in counteracting established sepsis (137). Contrarily, genetic modifications and drugs commonly take effect prior to induction of experimental sepsis in mice. Therefore, observations are only partly translatable to the patient situation.

#### Physiological Differences Between Mice and Humans

Most prominently, humans and mice differ considerably in their circulating immune cell composition. In humans, neutrophils constitute the most abundant leukocyte subpopulation (40–70%), whereas mice show up to 84% lymphocytes (193), which may impact on the relative contributions of innate and adaptive immunity to host responses. As already mentioned, distinct receptor expression regarding TLRs, PARs and Fc receptors determines immune cell capacity of platelets. This makes it often difficult to translate results from animal models to the clinical situation. To overcome this problem, mice expressing human receptors have been generated. While this was successful in some cases (FcγRIIA) (194), other attempts failed so far to lead to functional receptor expression (PAR-1) (195, 196). However, the role of FcγRIIA was never addressed in a murine sepsis model. Furthermore, while LPS challenge yields similar inflammatory responses in mice and men, including cytokine production and lymphopenia, humans are more sensitive to LPS than mice, which necessitate the use of LPS concentrations in mouse models that surpass those required to induce septic shock in humans about 1,000–10,000-fold (197, 198).

Nevertheless, despite their pitfalls mouse models have been immensely helpful to further our understanding of the role of platelets in sepsis and have shed light on cause and effect of, e.g., thrombocytopenia.

## Conclusion

Although, it has been decades of research in sepsis, the gained knowledge did not lead to the discovery of an effective treatment approved in patients. Sepsis is a complex disease with multiple players resulting in a very heterogeneous patient population with different comorbidities, immune statuses, and susceptibilities to infection. Many strategies are currently under investigation to restore platelet count in sepsis patients. However, it is still not known whether thrombocytopenia is a cause or a consequence of sepsis severity and how platelets contribute to sepsis progression. Moreover, as platelet receptors regulate inflammatory hemostasis and infection in a stimulus- and organ-dependent manner, a better understanding of the receptors and the mechanisms involved is crucial for successful treatment. Another factor to take into consideration is the immune status of patients as different treatments might be required based on the immune profile of the patients. While patients with SIRS would benefit from an anti-inflammatory therapy, immune-suppressed patients might benefit from an immuno-adjuvant therapy. Anti-platelet therapies, in particular aspirin, seem promising in experimental sepsis, however the risk of bleeding has to be closely monitored. Currently two clinical trials address the role of aspirin in patients and the outcome of these studies are expected to further clarify the beneficial use of aspirin in septic patients.

The use of mouse models shed light on new mechanisms in sepsis, however many factors limit the translation to the human setting. One major concern when targeting platelets is their dual role in inflammation and hemostasis. Platelets are not only pro-inflammatory cells but they also contribute to the resolution of inflammation and tissue repair. Most of the studies performed in mice use wild-type mice that lack FcγRIIA on platelets, one of the major receptors on platelets regulating pathogen-mediated activation, raising the question if FcγRIIA transgenic mice are required to investigate infection-mediated sepsis in mice. Moreover, comorbidities, age, and other factors might need to be taken into consideration in experimental models to reflect the clinical profile of the patients. In this context, animal models associated with other comorbidities may provide a better understanding of sepsis pathophysiology. A deeper knowledge of the role of platelet receptors in sepsis along with randomized clinical trials will determine the beneficial potential of different anti-platelet therapies in patients.

## Author Contributions

All authors listed have made a substantial, direct and intellectual contribution to the work, and approved it for publication.

### Conflict of Interest Statement

The authors declare that the research was conducted in the absence of any commercial or financial relationships that could be construed as a potential conflict of interest.

## References

[1] Singer M Deutschman CS Seymour CW Shankar-Hari M Annane D Bauer M The Third International consensus definitions for sepsis and septic shock (Sepsis-3). JAMA. (2016) 315:801–10. 10.1001/jama.2016.0287 26903338 PMC4968574

[2] Hotchkiss RS Monneret G Payen D. Sepsis-induced immunosuppression: from cellular dysfunctions to immunotherapy. Nat Rev Immunol. (2013) 13:862–74. 10.1038/nri3552 24232462 PMC4077177

[3] Martin GS. Sepsis, severe sepsis and septic shock: changes in incidence, pathogens and outcomes. Expert Rev Anti Infect Ther. (2012) 10:701–6. 10.1586/eri.12.50 22734959 PMC3488423

[4] Angus DC van der Poll T Severe sepsis and septic shock. N Engl J Med. (2013) 369:2063 10.1056/NEJMra1208623 24256390

[5] Kawai T Akira S. Toll-like receptors and their crosstalk with other innate receptors in infection and immunity. Immunity. (2011) 34:637–50. 10.1016/j.immuni.2011.05.006 21616434

[6] Hoesel B Schmid JA. The complexity of NF-kappaB signaling in inflammation and cancer. Mol Cancer. (2013) 12:86. 10.1186/1476-4598-12-86 23915189 PMC3750319

[7] Greenlee-Wacker MC. Clearance of apoptotic neutrophils and resolution of inflammation. Immunol Rev. (2016) 273:357–70. 10.1111/imr.12453 27558346 PMC5000862

[8] Mayr FB Yende S Angus DC. Epidemiology of severe sepsis. Virulence. (2014) 5:4–11. 10.4161/viru.27372 24335434 PMC3916382

[9] Martin GS Mannino DM Eaton S Moss M. The epidemiology of sepsis in the United States from 1979 through 2000. N Engl J Med. (2003) 348:1546–54. 10.1056/NEJMoa022139 12700374

[10] Gupta S Sakhuja A Kumar G McGrath E Nanchal RS Kashani KB. Culture-negative severe sepsis: nationwide trends and outcomes. Chest. (2016) 150:1251–9. 10.1016/j.chest.2016.08.1460 27615024

[11] Opal SM van der Poll T. Endothelial barrier dysfunction in septic shock. J Intern Med. (2015) 277:277–93. 10.1111/joim.12331 25418337

[12] Baskurt OK Gelmont D Meiselman HJ. Red blood cell deformability in sepsis. Am J Respir Crit Care Med. (1998) 157:421–7. 10.1164/ajrccm.157.2.9611103 9476853

[13] Pena OM Hancock DG Lyle NH Linder A Russell JA Xia J. An endotoxin tolerance signature predicts sepsis and organ dysfunction at initial clinical presentation. EBioMedicine. (2014) 1:64–71. 10.1016/j.ebiom.2014.10.003 25685830 PMC4326653

[14] Girardot T Rimmele T Venet F Monneret G. Apoptosis-induced lymphopenia in sepsis and other severe injuries. Apoptosis. (2017) 22:295–305. 10.1007/s10495-016-1325-3 27812767

[15] Boomer JS To K Chang KC Takasu O Osborne DF Walton AH. Immunosuppression in patients who die of sepsis and multiple organ failure. JAMA. (2011) 306:2594–605. 10.1001/jama.2011.1829 22187279 PMC3361243

[16] Ho J Yu J Wong SH Zhang L Liu X Wong WT. Autophagy in sepsis: degradation into exhaustion? Autophagy. (2016) 12:1073–82. 10.1080/15548627.2016.1179410 27172163 PMC4990998

[17] Rittirsch D Flierl MA Ward PA. Harmful molecular mechanisms in sepsis. Nat Rev Immunol. (2008) 8:776–87. 10.1038/nri2402 18802444 PMC2786961

[18] Beristain-Covarrubias N Perez-Toledo M Flores-Langarica A Zuidscherwoude M Hitchcock JR Channell WM *Salmonella*-induced thrombi in mice develop asynchronously in the spleen and liver and are not effective bacterial traps. Blood. (2018) 133:600–4. 10.1182/blood-2018-08-867267 30401709 PMC6474721

[19] Engelmann B Massberg S. Thrombosis as an intravascular effector of innate immunity. Nat Rev Immunol. (2013) 13:34–45. 10.1038/nri3345 23222502

[20] Levi M Lowenberg EC. Thrombocytopenia in critically ill patients. Semin Thromb Hemost. (2008) 34:417–24. 10.1055/s-0028-1092871 18956281

[21] Shibazaki M Nakamura M Endo Y. Biphasic, organ-specific, and strain-specific accumulation of platelets induced in mice by a lipopolysaccharide from *Escherichia coli* and its possible involvement in shock. Infect Immun. (1996) 64:5290–4. 8945579 10.1128/iai.64.12.5290-5294.1996 PMC174521

[22] Sigurdsson GH Christenson JT el-Rakshy MB Sadek S. Intestinal platelet trapping after traumatic and septic shock. An early sign of sepsis and multiorgan failure in critically ill patients? Crit Care Med. (1992) 20:458–67. 10.1097/00003246-199204000-00005 1559357

[23] Andonegui G Kerfoot SM McNagny K Ebbert KV Patel KD Kubes P. Platelets express functional Toll-like receptor-4. Blood. (2005) 106:2417–23. 10.1182/blood-2005-03-0916 15961512

[24] Levi M Ten Cate H. Disseminated intravascular coagulation. N Engl J Med. (1999) 341:586–92. 10.1056/NEJM199908193410807 10451465

[25] Claushuis TA van Vught LA Scicluna BP Wiewel MA Klein Klouwenberg PM Hoogendijk AJ. Thrombocytopenia is associated with a dysregulated host response in critically ill sepsis patients. Blood. (2016) 127:3062–72. 10.1182/blood-2015-11-680744 26956172

[26] Tsirigotis P Chondropoulos S Frantzeskaki F Stamouli M Gkirkas K Bartzeliotou A. Thrombocytopenia in critically ill patients with severe sepsis/septic shock: prognostic value and association with a distinct serum cytokine profile. J Crit Care. (2016) 32:9–15. 10.1016/j.jcrc.2015.11.010 26726794

[27] Mavrommatis AC Theodoridis T Orfanidou A Roussos C Christopoulou-Kokkinou V Zakynthinos S. Coagulation system and platelets are fully activated in uncomplicated sepsis. Crit Care Med. (2000) 28:451–7. 10.1097/00003246-200002000-00027 10708182

[28] Baughman RP Lower EE Flessa HC Tollerud DJ. Thrombocytopenia in the intensive care unit. Chest. (1993) 104:1243–7. 10.1378/chest.104.4.1243 8404200

[29] Vincent JL Zhang H Szabo C Preiser JC. Effects of nitric oxide in septic shock. Am J Respir Crit Care Med. (2000) 161:1781–5. 10.1164/ajrccm.161.6.9812004 10852744

[30] Aird WC. The role of the endothelium in severe sepsis and multiple organ dysfunction syndrome. Blood. (2003) 101:3765–77. 10.1182/blood-2002-06-1887 12543869

[31] Haak BW Wiersinga WJ. The role of the gut microbiota in sepsis. Lancet Gastroenterol Hepatol. (2017) 2:135–43. 10.1016/S2468-1253(16)30119-4 28403983

[32] Zarbock A Ley K. The role of platelets in acute lung injury (ALI). Front Biosci. (2009) 14:150–8. 10.2741/3236 19273059 PMC2745111

[33] Yan J Li S Li S. The role of the liver in sepsis. Int Rev Immunol. (2014) 33:498–510. 10.3109/08830185.2014.889129 24611785 PMC4160418

[34] Gomez H Kellum JA. Sepsis-induced acute kidney injury. Curr Opin Crit Care. (2016) 22:546–53. 10.1097/MCC.0000000000000356 27661757 PMC5654474

[35] Sonneville R Verdonk F Rauturier C Klein IF Wolff M Annane D. Understanding brain dysfunction in sepsis. Ann Intensive Care. (2013) 3:15. 10.1186/2110-5820-3-15 23718252 PMC3673822

[36] Kral JB Schrottmaier WC Salzmann M Assinger A. Platelet Interaction with Innate Immune Cells. Transfus Med Hemother. (2016) 43:78–88. 10.1159/000444807 27226790 PMC4872052

[37] Schrottmaier WC Kral JB Badrnya S Assinger A. Aspirin and P2Y12 Inhibitors in platelet-mediated activation of neutrophils and monocytes. Thromb Haemost. (2015) 114:478–89. 10.1160/TH14-11-0943 25904241

[38] Aslam R Speck ER Kim M Crow AR Bang KW Nestel FP. Platelet Toll-like receptor expression modulates lipopolysaccharide-induced thrombocytopenia and tumor necrosis factor-alpha production *in vivo*. Blood. (2006) 107:637–41. 10.1182/blood-2005-06-2202 16179373

[39] Handtke S Steil L Greinacher A Thiele T. Toward the relevance of platelet subpopulations for transfusion medicine. Front Med. (2018) 5:17. 10.3389/fmed.2018.00017 29459897 PMC5807390

[40] Koupenova M Mick E Mikhalev E Benjamin EJ Tanriverdi K Freedman JE. Sex differences in platelet toll-like receptors and their association with cardiovascular risk factors. Arterioscler Thromb Vasc Biol. (2015) 35:1030–7. 10.1161/ATVBAHA.114.304954 25657311 PMC4376646

[41] Burkhart JM Vaudel M Gambaryan S Radau S Walter U Martens L. The first comprehensive and quantitative analysis of human platelet protein composition allows the comparative analysis of structural and functional pathways. Blood. (2012) 120:e73–82. 10.1182/blood-2012-04-416594 22869793

[42] Cognasse F Nguyen KA Damien P McNicol A Pozzetto B Hamzeh-Cognasse H. The inflammatory role of platelets via their TLRs and siglec receptors. Front Immunol. (2015) 6:83. 10.3389/fimmu.2015.00083 25784910 PMC4345914

[43] Zeiler M Moser M Mann M. Copy number analysis of the murine platelet proteome spanning the complete abundance range. Mol Cell Proteomics. (2014) 13:3435–45. 10.1074/mcp.M114.038513 25205226 PMC4256495

[44] O'Neill LA. The interleukin-1 receptor/Toll-like receptor superfamily: 10 years of progress. Immunol Rev. (2008) 226:10–8. 10.1111/j.1600-065X.2008.00701.x 19161412

[45] Beaulieu LM Lin E Morin KM Tanriverdi K Freedman JE. Regulatory effects of TLR2 on megakaryocytic cell function. Blood. (2011) 117:5963–74. 10.1182/blood-2010-09-304949 21454454 PMC3112041

[46] Blair P Rex S Vitseva O Beaulieu L Tanriverdi K Chakrabarti S. Stimulation of Toll-like receptor 2 in human platelets induces a thromboinflammatory response through activation of phosphoinositide 3-kinase. Circ Res. (2009) 104:346–54. 10.1161/CIRCRESAHA.108.185785 19106411 PMC2732983

[47] Assinger A Buchberger E Laky M Esfandeyari A Brostjan C Volf I. Periodontopathogens induce soluble P-selectin release by endothelial cells and platelets. Thromb Res. (2011) 127:e20–6. 10.1016/j.thromres.2010.10.023 21106229

[48] Koupenova M Vitseva O MacKay CR Beaulieu LM Benjamin EJ Mick E. Platelet-TLR7 mediates host survival and platelet count during viral infection in the absence of platelet-dependent thrombosis. Blood. (2014) 124:791–802. 10.1182/blood-2013-11-536003 24755410 PMC4118487

[49] Thon JN Peters CG Machlus KR Aslam R Rowley J Macleod H. T granules in human platelets function in TLR9 organization and signaling. J Cell Biol. (2012) 198:561–74. 10.1083/jcb.201111136 22908309 PMC3514030

[50] Clark SR Ma AC Tavener SA McDonald B Goodarzi Z Kelly MM. Platelet TLR4 activates neutrophil extracellular traps to ensnare bacteria in septic blood. Nat Med. (2007) 13:463–9. 10.1038/nm1565 17384648

[51] Semple JW Aslam R Kim M Speck ER Freedman J. Platelet-bound lipopolysaccharide enhances Fc receptor-mediated phagocytosis of IgG-opsonized platelets. Blood. (2007) 109:4803–5. 10.1182/blood-2006-12-062695 17299089

[52] Bentala H Verweij WR Huizinga-Van der Vlag A van Loenen-Weemaes AM Meijer DK Poelstra K. Removal of phosphate from lipid A as a strategy to detoxify lipopolysaccharide. Shock. (2002) 18:561–6. 10.1097/00024382-200212000-00013 12462566

[53] Schromm AB Brandenburg K Loppnow H Zahringer U Rietschel ET Carroll SF. The charge of endotoxin molecules influences their conformation and IL-6-inducing capacity. J Immunol. (1998) 161:5464–71. 9820522

[54] Yang WH Heithoff DM Aziz PV Haslund-Gourley B Westman JS Narisawa S. Accelerated aging and clearance of host anti-inflammatory enzymes by discrete pathogens fuels sepsis. Cell Host Microbe. (2018) 24:500–513 e5. 10.1016/j.chom.2018.09.011 30308156 PMC6223661

[55] Grewal PK Aziz PV Uchiyama S Rubio GR Lardone RD Le D. Inducing host protection in pneumococcal sepsis by preactivation of the Ashwell-Morell receptor. Proc Natl Acad Sci USA. (2013) 110:20218–23. 10.1073/pnas.1313905110 24284176 PMC3864324

[56] Claushuis TAM Van Der Veen AIP Horn J Schultz MJ Houtkooper RH Van 't Veer C. Platelet Toll-like receptor expression and activation induced by lipopolysaccharide and sepsis. Platelets. (2018) 30:296–304. 10.1080/09537104.2018.1445841 29528268

[57] Boukour S Masse JM Benit L Dubart-Kupperschmitt A Cramer EM. Lentivirus degradation and DC-SIGN expression by human platelets and megakaryocytes. J Thromb Haemost. (2006) 4:426–35. 10.1111/j.1538-7836.2006.01749.x 16420576

[58] Tsicopoulos A Joseph M. The role of CD23 in allergic disease. Clin Exp Allergy. (2000) 30:602–5. 10.1046/j.1365-2222.2000.00871.x 10792350

[59] Joseph M Gounni AS Kusnierz JP Vorng H Sarfati M Kinet JP. Expression and functions of the high-affinity IgE receptor on human platelets and megakaryocyte precursors. Eur J Immunol. (1997) 27:2212–8. 10.1002/eji.1830270914 9341761

[60] Klouche M Klinger MH Kuhnel W Wilhelm D. Endocytosis, storage, and release of IgE by human platelets: differences in patients with type I allergy and nonatopic subjects. J Allergy Clin Immunol. (1997) 100:235–41. 10.1016/S0091-6749(97)70230-6 9275146

[61] Philpott DJ Sorbara MT Robertson SJ Croitoru K Girardin SE. NOD proteins: regulators of inflammation in health and disease. Nat Rev Immunol. (2014) 14:9–23. 10.1038/nri3565 24336102

[62] Zhang S Zhang S Hu L Zhai L Xue R Ye J. Nucleotide-binding oligomerization domain 2 receptor is expressed in platelets and enhances platelet activation and thrombosis. Circulation. (2015) 131:1160–70. 10.1161/CIRCULATIONAHA.114.013743 25825396 PMC4382913

[63] Plummer C Wu H Kerrigan SW Meade G Cox D Ian Douglas CW. A serine-rich glycoprotein of Streptococcus sanguis mediates adhesion to platelets via GPIb. Br J Haematol. (2005) 129:101–9. 10.1111/j.1365-2141.2005.05421.x 15801962

[64] Hartleib J Kohler N Dickinson RB Chhatwal GS Sixma JJ Hartford OM. Protein A is the von Willebrand factor binding protein on *Staphylococcus aureus*. Blood. (2000) 96:2149–56. 10979960

[65] Bennett JS. Structure and function of the platelet integrin alphaIIbbeta3. J Clin Invest. (2005) 115:3363–9. 10.1172/JCI26989 16322781 PMC1297263

[66] Brennan MP Loughman A Devocelle M Arasu S Chubb AJ Foster TJ. Elucidating the role of *Staphylococcus epidermidis* serine-aspartate repeat protein G in platelet activation. J Thromb Haemost. (2009) 7:1364–72. 10.1111/j.1538-7836.2009.03495.x 19486275

[67] Coburn J Leong JM Erban JK. Integrin alpha IIb beta 3 mediates binding of the Lyme disease agent *Borrelia burgdorferi* to human platelets. Proc Natl Acad Sci USA. (1993) 90:7059–63. 10.1073/pnas.90.15.7059 8394007 PMC47075

[68] Siboo IR Cheung AL Bayer AS Sullam PM. Clumping factor A mediates binding of *Staphylococcus aureus* to human platelets. Infect Immun. (2001) 69:3120–7. 10.1128/IAI.69.5.3120-3127.2001 11292731 PMC98267

[69] Kahn ML Zheng YW Huang W Bigornia V Zeng D Moff S. A dual thrombin receptor system for platelet activation. Nature. (1998) 394:690–4. 10.1038/29325 9716134

[70] Qian K Xie F Gibson AW Edberg JC Kimberly RP Wu J. Functional expression of IgA receptor FcalphaRI on human platelets. J Leukoc Biol. (2008) 84:1492–500. 10.1189/jlb.0508327 18784345 PMC2614599

[71] Hasegawa S Pawankar R Suzuki K Nakahata T Furukawa S Okumura K. Functional expression of the high affinity receptor for IgE (FcepsilonRI) in human platelets and its' intracellular expression in human megakaryocytes. Blood. (1999) 93:2543–51. 10194433

[72] Tomiyama Y Kunicki TJ Zipf TF Ford SB Aster RH. Response of human platelets to activating monoclonal antibodies: importance of Fc gamma RII (CD32) phenotype and level of expression. Blood. (1992) 80:2261–8. 1421396

[73] Arman M Krauel K. Human platelet IgG Fc receptor FcgammaRIIA in immunity and thrombosis. J Thromb Haemost. (2015) 13:893–908. 10.1111/jth.12905 25900780

[74] Riaz AH Tasma BE Woodman ME Wooten RM Worth RG. Human platelets efficiently kill IgG-opsonized *E. coli*. FEMS Immunol Med Microbiol. (2012) 65:78–83. 10.1111/j.1574-695X.2012.00945.x 22340259 PMC3342444

[75] Leon C Ravanat C Freund M Cazenave JP Gachet C. Differential involvement of the P2Y1 and P2Y12 receptors in platelet procoagulant activity. Arterioscler Thromb Vasc Biol. (2003) 23:1941–7. 10.1161/01.ATV.0000092127.16125.E6 12933533

[76] Salat A Bodingbauer G Boehm D Murabito M Tochkow E Sautner T. Changes of platelet surface antigens in patients suffering from abdominal septic shock. Thromb Res. (1999) 95:289–94. 10.1016/S0049-3848(99)00046-8 10527406

[77] Gawaz M Dickfeld T Bogner C Fateh-Moghadam S Neumann FJ. Platelet function in septic multiple organ dysfunction syndrome. Intensive Care Med. (1997) 23:379–85. 10.1007/s001340050344 9142575

[78] Layios N Delierneux C Hego A Huart J Gosset C Lecut C. Sepsis prediction in critically ill patients by platelet activation markers on ICU admission: a prospective pilot study. Intensive Care Med Exp. (2017) 5:32. 10.1186/s40635-017-0145-2 28699088 PMC5505890

[79] Montague SJ Delierneux C Lecut C Layios N Dinsdale RJ Lee CS. Soluble GPVI is elevated in injured patients: shedding is mediated by fibrin activation of GPVI. Blood Adv. (2018) 2:240–51. 10.1182/bloodadvances.2017011171 29437639 PMC5812322

[80] Laursen MA Larsen JB Hvas AM. Platelet function in disseminated intravascular coagulation: a systematic review. Platelets. (2018) 29:238–48. 10.1080/09537104.2018.1442567 29517400

[81] Woth G Varga A Ghosh S Krupp M Kiss T Bogar L. Platelet aggregation in severe sepsis. J Thromb Thrombolysis. (2011) 31:6–12. 10.1007/s11239-010-0486-0 20455008

[82] Yaguchi A Lobo FL Vincent JL Pradier O. Platelet function in sepsis. J Thromb Haemost. (2004) 2:2096–102. 10.1111/j.1538-7836.2004.01009.x 15613012

[83] Vincent JL Yagushi A Pradier O. Platelet function in sepsis. Crit Care Med. (2002) 30:S313–7. 10.1097/00003246-200205001-00022 12004253

[84] Gawaz M Fateh-Moghadam S Pilz G Gurland HJ Werdan K Platelet activation and interaction with leucocytes in patients with sepsis or multiple organ failure. Eur J Clin Invest. (1995) 25:843–51. 10.1111/j.1365-2362.1995.tb01694.x 8582450

[85] Rondina MT Carlisle M Fraughton T Brown SM Miller RR III Harris ES. Platelet-monocyte aggregate formation and mortality risk in older patients with severe sepsis and septic shock. J Gerontol A Biol Sci Med Sci. (2015) 70:225–31. 10.1093/gerona/glu082 24917177 PMC4366600

[86] Arman M Krauel K Tilley DO Weber C Cox D Greinacher A. Amplification of bacteria-induced platelet activation is triggered by FcgammaRIIA, integrin alphaIIbbeta3, and platelet factor 4. Blood. (2014) 123:3166–74. 10.1182/blood-2013-11-540526 24642751 PMC4023422

[87] Hamzeh-Cognasse H Damien P Chabert A Pozzetto B Cognasse F Garraud O. Platelets and infections - complex interactions with bacteria. Front Immunol. (2015) 6:82. 10.3389/fimmu.2015.00082 25767472 PMC4341565

[88] Cox D Kerrigan SW Watson SP. Platelets and the innate immune system: mechanisms of bacterial-induced platelet activation. J Thromb Haemost. (2011) 9:1097–107. 10.1111/j.1538-7836.2011.04264.x 21435167

[89] Ghuman H Shepherd-Roberts A Watson S Zuidscherwoude M Watson SP Voelz K. Mucor circinelloides induces platelet aggregation through integrin alphaIIbbeta3 and FcgammaRIIA. Platelets. (2018) 30:256–63. 10.1080/09537104.2017.1420152 29297721

[90] Svensson L Baumgarten M Morgelin M Shannon O. Platelet activation by *Streptococcus pyogenes* leads to entrapment in platelet aggregates, from which bacteria subsequently escape. Infect Immun. (2014) 82:4307–14. 10.1128/IAI.02020-14 25069984 PMC4187850

[91] Markiewski MM DeAngelis RA Lambris JD. Complexity of complement activation in sepsis. J Cell Mol Med. (2008) 12:2245–54. 10.1111/j.1582-4934.2008.00504.x 18798865 PMC2673539

[92] Vanderschueren S De Weerdt A Malbrain M Vankersschaever D Frans E Wilmer A. Thrombocytopenia and prognosis in intensive care. Crit Care Med. (2000) 28:1871–6. 10.1097/00003246-200006000-00031 10890635

[93] Sharma B Sharma M Majumder M Steier W Sangal A Kalawar M. Thrombocytopenia in septic shock patients–a prospective observational study of incidence, risk factors and correlation with clinical outcome. Anaesth Intensive Care. (2007) 35:874–80. 10.1177/0310057X0703500604 18084977

[94] Hui P Cook DJ Lim W Fraser GA Arnold DM. The frequency and clinical significance of thrombocytopenia complicating critical illness: a systematic review. Chest. (2011) 139:271–8. 10.1378/chest.10-2243 21071526

[95] Thiolliere F Serre-Sapin AF Reignier J Benedit M Constantin JM Lebert C. Epidemiology and outcome of thrombocytopenic patients in the intensive care unit: results of a prospective multicenter study. Intensive Care Med. (2013) 39:1460–8. 10.1007/s00134-013-2963-3 23740274

[96] Venkata C Kashyap R Farmer JC Afessa B. Thrombocytopenia in adult patients with sepsis: incidence, risk factors, and its association with clinical outcome. J Intensive Care. (2013) 1:9. 10.1186/2052-0492-1-9 25810916 PMC4373028

[97] Akca S Haji-Michael P de Mendonca A Suter P Levi M Vincent JL. Time course of platelet counts in critically ill patients. Crit Care Med. (2002) 30:753–6. 10.1097/00003246-200204000-00005 11940740

[98] Drews RE Weinberger SE. Thrombocytopenic disorders in critically ill patients. Am J Respir Crit Care Med. (2000) 162:347–51. 10.1164/ajrccm.162.2.ncc3-00 10934051

[99] Dewitte A Lepreux S Villeneuve J Rigothier C Combe C Ouattara A Blood platelets and sepsis pathophysiology: A new therapeutic prospect in critical ill patients? Ann Intensive Care. (2017) 7:115 10.1186/s13613-017-0337-7 29192366 PMC5709271

[100] Vincent JL Castro P Hunt BJ Jorres A Praga M Rojas-Suarez J. Thrombocytopenia in the ICU: disseminated intravascular coagulation and thrombotic microangiopathies-what intensivists need to know. Crit Care. (2018) 22:158. 10.1186/s13054-018-2073-2 29895296 PMC5998546

[101] Strauss R Wehler M Mehler K Kreutzer D Koebnick C Hahn EG. Thrombocytopenia in patients in the medical intensive care unit: bleeding prevalence, transfusion requirements, and outcome. Crit Care Med. (2002) 30:1765–71. 10.1097/00003246-200208000-00015 12163790

[102] Koyama K Katayama S Muronoi T Tonai K Goto Y Koinuma T. Time course of immature platelet count and its relation to thrombocytopenia and mortality in patients with sepsis. PLoS ONE. (2018) 13:e0192064. 10.1371/journal.pone.0192064 29381746 PMC5790259

[103] De Blasi RA Cardelli P Costante A Sandri M Mercieri M Arcioni R. Immature platelet fraction in predicting sepsis in critically ill patients. Intensive Care Med. (2013) 39:636–43. 10.1007/s00134-012-2725-7 23093245

[104] Puskarich MA Cornelius DC Bandyopadhyay S McCalmon M Tramel R Dale WD. Phosphatidylserine expressing platelet microparticle levels at hospital presentation are decreased in sepsis non-survivors and correlate with thrombocytopenia. Thromb Res. (2018) 168:138–44. 10.1016/j.thromres.2018.06.017 30064685 PMC6310160

[105] Elaskalani O Abdol Razak NB Metharom P. Neutrophil extracellular traps induce aggregation of washed human platelets independently of extracellular DNA and histones. Cell Commun Signal. (2018) 16:24. 10.1186/s12964-018-0235-0 29843771 PMC5975482

[106] Hu H Armstrong PC Khalil E Chen YC Straub A Li M. GPVI and GPIbalpha mediate staphylococcal superantigen-like protein 5 (SSL5) induced platelet activation and direct toward glycans as potential inhibitors. PLoS ONE. (2011) 6:e19190. 10.1371/journal.pone.0019190 21552524 PMC3084272

[107] Kraemer BF Campbell RA Schwertz H Franks ZG Vieira de Abreu A Grundler K. Bacteria differentially induce degradation of Bcl-xL, a survival protein, by human platelets. Blood. (2012) 120:5014–20. 10.1182/blood-2012-04-420661 23086749 PMC3525025

[108] Kapur R Heitink-Polle KM Porcelijn L Bentlage AE Bruin MC Visser R. C-reactive protein enhances IgG-mediated phagocyte responses and thrombocytopenia. Blood. (2015) 125:1793–802. 10.1182/blood-2014-05-579110 25548320

[109] Johansson D Rasmussen M Inghammar M. Thrombocytopenia in bacteraemia and association with bacterial species. Epidemiol Infect. (2018) 146:1312–7. 10.1017/S0950268818001206 29759089 PMC9134296

[110] Stark RJ Aghakasiri N Rumbaut RE. Platelet-derived Toll-like receptor 4 (Tlr-4) is sufficient to promote microvascular thrombosis in endotoxemia. PLoS ONE. (2012) 7:e41254. 10.1371/journal.pone.0041254 22911769 PMC3401143

[111] Semeraro F Ammollo CT Morrissey JH Dale GL Friese P Esmon NL. Extracellular histones promote thrombin generation through platelet-dependent mechanisms: involvement of platelet TLR2 and TLR4. Blood. (2011) 118:1952–61. 10.1182/blood-2011-03-343061 21673343 PMC3158722

[112] Ito T. PAMPs and DAMPs as triggers for DIC. J Intensive Care. (2014) 2:67. 10.1186/s40560-014-0065-0 25705424 PMC4336279

[113] Vogel S Bodenstein R Chen Q Feil S Feil R Rheinlaender J. Platelet-derived HMGB1 is a critical mediator of thrombosis. J Clin Invest. (2015) 125:4638–54. 10.1172/JCI81660 26551681 PMC4665785

[114] Li MF Li XL Fan KL Yu YY Gong J Geng SY. Platelet desialylation is a novel mechanism and a therapeutic target in thrombocytopenia during sepsis: an open-label, multicenter, randomized controlled trial. J Hematol Oncol. (2017) 10:104. 10.1186/s13045-017-0476-1 28494777 PMC5426054

[115] Kullaya V de Jonge MI Langereis JD van der Gaast-de Jongh CE Bull C Adema GJ. Desialylation of platelets by pneumococcal neuraminidase A induces ADP-dependent platelet hyperreactivity. Infect Immun. (2018) 86:e00213–18. 10.1128/IAI.00213-18 30037798 PMC6204724

[116] Ghosh TK Khan N Malik A. Platelet auto-antibodies in septicaemic patients. Indian J Pathol Microbiol. (1999) 42:31–5. 10420682

[117] Stephan F Hollande J Richard O Cheffi A Maier-Redelsperger M Flahault A. Thrombocytopenia in a surgical ICU. Chest. (1999) 115:1363–70. 10.1378/chest.115.5.1363 10334154

[118] Maharaj S Chang S. Anti-PF4/heparin antibodies are increased in hospitalized patients with bacterial sepsis. Thromb Res. (2018) 171:111–3. 10.1016/j.thromres.2018.09.060 30273811

[119] Cloutier N Allaeys I Marcoux G Machlus KR Mailhot B Zufferey A. Platelets release pathogenic serotonin and return to circulation after immune complex-mediated sequestration. Proc Natl Acad Sci USA. (2018) 115:E1550–9. 10.1073/pnas.1720553115 29386381 PMC5816207

[120] Aird WC. The hematologic system as a marker of organ dysfunction in sepsis. Mayo Clin Proc. (2003) 78:869–81. 10.4065/78.7.869 12839083

[121] Jimenez-Alcazar M Rangaswamy C Panda R Bitterling J Simsek YJ Long AT. Host DNases prevent vascular occlusion by neutrophil extracellular traps. Science. (2017) 358:1202–6. 10.1126/science.aam8897 29191910

[122] Muronoi T Koyama K Nunomiya S Lefor AK Wada M Koinuma T. Immature platelet fraction predicts coagulopathy-related platelet consumption and mortality in patients with sepsis. Thromb Res. (2016) 144:169–75. 10.1016/j.thromres.2016.06.002 27380496

[123] Lupia E Goffi A Bosco O Montrucchio G. Thrombopoietin as biomarker and mediator of cardiovascular damage in critical diseases. Mediators Inflamm. (2012) 2012:390892. 10.1155/2012/390892 22577249 PMC3337636

[124] Francois B Trimoreau F Vignon P Fixe P Praloran V Gastinne H. Thrombocytopenia in the sepsis syndrome: role of hemophagocytosis and macrophage colony-stimulating factor. Am J Med. (1997) 103:114–20. 10.1016/S0002-9343(97)00136-8 9274894

[125] Losche W Boettel J Kabisch B Winning J Claus RA Bauer M. Do aspirin and other antiplatelet drugs reduce the mortality in critically ill patients? Thrombosis. (2012) 2012:720254. 10.1155/2012/720254 22110915 PMC3216368

[126] Winning J Neumann J Kohl M Claus RA Reinhart K Bauer M. Antiplatelet drugs and outcome in mixed admissions to an intensive care unit. Crit Care Med. (2010) 38:32–7. 10.1097/CCM.0b013e3181b4275c 19770746

[127] Thomas MR Outteridge SN Ajjan RA Phoenix F Sangha GK Faulkner RE. Platelet P2Y12 inhibitors reduce systemic inflammation and its prothrombotic effects in an experimental human model. Arterioscler Thromb Vasc Biol. (2015) 35:2562–70. 10.1161/ATVBAHA.115.306528 26515417 PMC4663676

[128] Sossdorf M Otto GP Boettel J Winning J Losche W. Benefit of low-dose aspirin and non-steroidal anti-inflammatory drugs in septic patients. Crit Care. (2013) 17:402. 10.1186/cc11886 23294562 PMC4056751

[129] Eisen DP Reid D McBryde ES. Acetyl salicylic acid usage and mortality in critically ill patients with the systemic inflammatory response syndrome and sepsis. Crit Care Med. (2012) 40:1761–7. 10.1097/CCM.0b013e318246b9df 22610182

[130] Rayes J Watson SP Nieswandt B. Functional significance of the platelet immune receptors GPVI and CLEC-2. J Clin Invest. (2019) 129:12–23. 10.1172/JCI122955 30601137 PMC6307936

[131] Zhang G Han J Welch EJ Ye RD Voyno-Yasenetskaya TA Malik AB. Lipopolysaccharide stimulates platelet secretion and potentiates platelet aggregation via TLR4/MyD88 and the cGMP-dependent protein kinase pathway. J Immunol. (2009) 182:7997–8004. 10.4049/jimmunol.0802884 19494325 PMC2787095

[132] de Stoppelaar SF Claushuis TA Jansen MP Hou B Roelofs JJ van 't Veer C. The role of platelet MyD88 in host response during gram-negative sepsis. J Thromb Haemost. (2015) 13:1709–20. 10.1111/jth.13048 26178922

[133] Ohtaki Y Shimauchi H Yokochi T Takada H Endo Y. *In vivo* platelet response to lipopolysaccharide in mice: proposed method for evaluating new antiplatelet drugs. Thromb Res. (2002) 108:303–9. 10.1016/S0049-3848(03)00092-6 12676190

[134] Corral J Yelamos J Hernandez-Espinosa D Monreal Y Mota R Arcas I. Role of lipopolysaccharide and cecal ligation and puncture on blood coagulation and inflammation in sensitive and resistant mice models. Am J Pathol. (2005) 166:1089–98. 10.1016/S0002-9440(10)62329-2 15793289 PMC1602389

[135] Zhao L Ohtaki Y Yamaguchi K Matsushita M Fujita T Yokochi T. LPS-induced platelet response and rapid shock in mice: contribution of O-antigen region of LPS and involvement of the lectin pathway of the complement system. Blood. (2002) 100:3233–9. 10.1182/blood-2002-01-0252 12384422

[136] Shibazaki M Kawabata Y Yokochi T Nishida A Takada H Endo Y. Complement-dependent accumulation and degradation of platelets in the lung and liver induced by injection of lipopolysaccharides. Infect Immun. (1999) 67:5186–91. 10496894 10.1128/iai.67.10.5186-5191.1999 PMC96869

[137] Stortz JA Raymond SL Mira JC Moldawer LL Mohr AM Efron PA. Murine models of sepsis and trauma: can we bridge the gap? ILAR J. (2017) 58:90–105. 10.1093/ilar/ilx007 28444204 PMC5886315

[138] Zhang S Zhang S Rahman M Herwald H Thorlacius H. Streptococcal M1 protein-induced lung injury is independent of platelets in mice. Shock. (2011) 35:86–91. 10.1097/SHK.0b013e3181ea4476 20577151

[139] von Asmuth EJ Maessen JG van der Linden CJ Buurman WA. Tumour necrosis factor alpha (TNF-alpha) and interleukin 6 in a zymosan-induced shock model. Scand J Immunol. (1990) 32:313–9. 10.1111/j.1365-3083.1990.tb02925.x 2237286

[140] Xiang B Zhang G Guo L Li XA Morris AJ Daugherty A. Platelets protect from septic shock by inhibiting macrophage-dependent inflammation via the cyclooxygenase 1 signalling pathway. Nat Commun. (2013) 4:2657. 10.1038/ncomms3657 24150174 PMC4217311

[141] de Stoppelaar SF van 't Veer C Claushuis TA Albersen BJ Roelofs JJ van der Poll T. Thrombocytopenia impairs host defense in gram-negative pneumonia-derived sepsis in mice. Blood. (2014) 124:3781–90. 10.1182/blood-2014-05-573915 25301709 PMC4263985

[142] Powers ME Becker RE Sailer A Turner JR Bubeck Wardenburg J. Synergistic action of *Staphylococcus aureus* alpha-toxin on platelets and myeloid lineage cells contributes to lethal sepsis. Cell Host Microbe. (2015) 17:775–87. 10.1016/j.chom.2015.05.011 26067604 PMC4642999

[143] Kahn F Hurley S Shannon O. Platelets promote bacterial dissemination in a mouse model of streptococcal sepsis. Microbes Infect. (2013) 15:669–76. 10.1016/j.micinf.2013.05.003 23711899

[144] van den Boogaard FE Schouten M de Stoppelaar SF Roelofs JJ Brands X Schultz MJ. Thrombocytopenia impairs host defense during murine *Streptococcus pneumoniae* pneumonia. Crit Care Med. (2015) 43:e75–83. 10.1097/CCM.0000000000000853 25627210

[145] Cross AS Opal SM Sadoff JC Gemski P. Choice of bacteria in animal models of sepsis. Infect Immun. (1993) 61:2741–7. 8514375 10.1128/iai.61.7.2741-2747.1993 PMC280916

[146] Feldman C Shaddock E. Epidemiology of lower respiratory tract infections in adults. Expert Rev Respir Med. (2018). 10.1080/17476348.2019.1555040 30518278

[147] Gow NAR Yadav B. Microbe profile: Candida albicans: a shape-changing, opportunistic pathogenic fungus of humans. Microbiology. (2017) 163:1145–7. 10.1099/mic.0.000499 28809155

[148] Qin Y Zhang L Xu Z Zhang J Jiang YY Cao Y. Innate immune cell response upon *Candida albicans* infection. Virulence. (2016) 7:512–26. 10.1080/21505594.2016.1138201 27078171 PMC5026795

[149] Holder IA Nathan P. Effect in mice of injection of viable *Candida albicans* and a cell-free sonic extract on circulating platelets. Infect Immun. (1973) 7:468–72. 4576682 10.1128/iai.7.3.468-472.1973 PMC422701

[150] Li JL Li G Jing XZ Li YF Ye QY Jia HH. Assessment of clinical sepsis-associated biomarkers in a septic mouse model. J Int Med Res. (2018) 46:2410–22. 10.1177/0300060518764717 29644918 PMC6023044

[151] Vardon Bounes F Memier V Marcaud M Jacquemin A Hamzeh-Cognasse H Garcia C. Platelet activation and prothrombotic properties in a mouse model of peritoneal sepsis. Sci Rep. (2018) 8:13536. 10.1038/s41598-018-31910-8 30201980 PMC6131186

[152] Dejager L Pinheiro I Dejonckheere E Libert C. Cecal ligation and puncture: the gold standard model for polymicrobial sepsis? Trends Microbiol. (2011) 19:198–208. 10.1016/j.tim.2011.01.001 21296575

[153] Buras JA Holzmann B Sitkovsky M. Animal models of sepsis: setting the stage. Nat Rev Drug Discov. (2005) 4:854–65. 10.1038/nrd1854 16224456

[154] Singer G Houghton J Rivera CA Anthoni C Granger DN. Role of LPS in the hepatic microvascular dysfunction elicited by cecal ligation and puncture in mice. J Hepatol. (2007) 47:799–806. 10.1016/j.jhep.2007.07.021 17935822 PMC2100413

[155] Remick DG Newcomb DE Bolgos GL Call DR. Comparison of the mortality and inflammatory response of two models of sepsis: lipopolysaccharide vs. cecal ligation and puncture. Shock. (2000) 13:110–6. 10.1097/00024382-200013020-00004 10670840

[156] Gentile LF Nacionales DC Lopez MC Vanzant E Cuenca A Szpila BE. Host responses to sepsis vary in different low-lethality murine models. PLoS ONE. (2014) 9:e94404. 10.1371/journal.pone.0094404 24788351 PMC4006924

[157] Claushuis TAM de Vos AF Nieswandt B Boon L Roelofs J de Boer OJ. Platelet glycoprotein VI aids in local immunity during pneumonia-derived sepsis caused by gram-negative bacteria. Blood. (2018) 131:864–76. 10.1182/blood-2017-06-788067 29187378

[158] Zarbock A Singbartl K Ley K. Complete reversal of acid-induced acute lung injury by blocking of platelet-neutrophil aggregation. J Clin Invest. (2006) 116:3211–9. 10.1172/JCI29499 17143330 PMC1679711

[159] Wuescher LM Takashima A Worth RG. A novel conditional platelet depletion mouse model reveals the importance of platelets in protection against *Staphylococcus aureus* bacteremia. J Thromb Haemost. (2015) 13:303–13. 10.1111/jth.12795 25418277 PMC4320667

[160] Camerer E Cornelissen I Kataoka H Duong DN Zheng YW Coughlin SR. Roles of protease-activated receptors in a mouse model of endotoxemia. Blood. (2006) 107:3912–21. 10.1182/blood-2005-08-3130 16434493 PMC1895289

[161] de Stoppelaar SF Van't Veer C van den Boogaard FE Nieuwland R Hoogendijk AJ de Boer OJ. Protease activated receptor 4 limits bacterial growth and lung pathology during late stage *Streptococcus pneumoniae* induced pneumonia in mice. Thromb Haemost. (2013) 110:582–92. 10.1160/TH13-01-0052 23783078

[162] de Stoppelaar SF Van't Veer C Roelofs JJ Claushuis TA de Boer OJ Tanck MW. Platelet and endothelial cell P-selectin are required for host defense against *Klebsiella pneumoniae*-induced pneumosepsis. J Thromb Haemost. (2015) 13:1128–38. 10.1111/jth.12893 25773400

[163] Asaduzzaman M Rahman M Jeppsson B Thorlacius H. P-selectin glycoprotein-ligand-1 regulates pulmonary recruitment of neutrophils in a platelet-independent manner in abdominal sepsis. Br J Pharmacol. (2009) 156:307–15. 10.1111/j.1476-5381.2008.00021.x 19154425 PMC2697831

[164] Rahman M Zhang S Chew M Syk I Jeppsson B Thorlacius H. Platelet shedding of CD40L is regulated by matrix metalloproteinase-9 in abdominal sepsis. J Thromb Haemost. (2013) 11:1385–98. 10.1111/jth.12273 23617547

[165] Asaduzzaman M Lavasani S Rahman M Zhang S Braun OO Jeppsson B. Platelets support pulmonary recruitment of neutrophils in abdominal sepsis. Crit Care Med. (2009) 37:1389–96. 10.1097/CCM.0b013e31819ceb71 19242347

[166] Yin H Stojanovic-Terpo A Xu W Corken A Zakharov A Qian F. Role for platelet glycoprotein Ib-IX and effects of its inhibition in endotoxemia-induced thrombosis, thrombocytopenia, and mortality. Arterioscler Thromb Vasc Biol. (2013) 33:2529–37. 10.1161/ATVBAHA.113.302339 24051142 PMC4043446

[167] Corken A Russell S Dent J Post SR Ware J. Platelet glycoprotein Ib-IX as a regulator of systemic inflammation. Arterioscler Thromb Vasc Biol. (2014) 34:996–1001. 10.1161/ATVBAHA.113.303113 24504734 PMC3991762

[168] Rayes J Lax S Wichaiyo S Watson SK Di Y Lombard S. The podoplanin-CLEC-2 axis inhibits inflammation in sepsis. Nat Commun. (2017) 8:2239. 10.1038/s41467-017-02402-6 29269852 PMC5740111

[169] Hitchcock JR Cook CN Bobat S Ross EA Flores-Langarica A Lowe KL eta l. Inflammation drives thrombosis after *Salmonella* infection via CLEC-2 on platelets. J Clin Invest. (2015) 125:4429–46. 10.1172/JCI79070 26571395 PMC4665792

[170] Claushuis TAM de Stoppelaar SF de Vos AF Grootemaat AE van der Wel NN Roelofs J Nbeal2 deficiency increases organ damage but does not affect host defense during gram-negative pneumonia-derived sepsis. Arterioscler Thromb Vasc Biol. (2018) 38:1772–84. 10.1161/ATVBAHA.118.311332 29930006 PMC13142737

[171] Hwaiz R Rahman M Zhang E Thorlacius H. Platelet secretion of CXCL4 is Rac1-dependent and regulates neutrophil infiltration and tissue damage in septic lung damage. Br J Pharmacol. (2015) 172:5347–59. 10.1111/bph.13325 26478565 PMC5341222

[172] Hwaiz R Rahman M Syk I Zhang E Thorlacius H. Rac1-dependent secretion of platelet-derived CCL5 regulates neutrophil recruitment via activation of alveolar macrophages in septic lung injury. J Leukoc Biol. (2015) 97:975–84. 10.1189/jlb.4A1214-603R 25717148

[173] Grommes J Alard JE Drechsler M Wantha S Morgelin M Kuebler WM. Disruption of platelet-derived chemokine heteromers prevents neutrophil extravasation in acute lung injury. Am J Respir Crit Care Med. (2012) 185:628–36. 10.1164/rccm.201108-1533OC 22246174 PMC3326286

[174] Kowalska MA Mahmud SA Lambert MP Poncz M Slungaard A Endogenous platelet factor 4 stimulates activated protein C generation *in vivo* and improves survival after thrombin or lipopolysaccharide challenge. Blood. (2007) 110:1903–5. 10.1182/blood-2007-03-081901 17540840 PMC1976343

[175] Duerschmied D Suidan GL Demers M Herr N Carbo C Brill A. Platelet serotonin promotes the recruitment of neutrophils to sites of acute inflammation in mice. Blood. (2013) 121:1008–15. 10.1182/blood-2012-06-437392 23243271 PMC3567335

[176] Zhou H Deng M Liu Y Yang C Hoffman R Zhou J. Platelet HMGB1 is required for efficient bacterial clearance in intra-abdominal bacterial sepsis in mice. Blood Adv. (2018) 2:638–48. 10.1182/bloodadvances.2017011817 29563120 PMC5873229

[177] Freishtat RJ Natale J Benton AS Cohen J Sharron M Wiles AA. Sepsis alters the megakaryocyte-platelet transcriptional axis resulting in granzyme B-mediated lymphotoxicity. Am J Respir Crit Care Med. (2009) 179:467–73. 10.1164/rccm.200807-1085OC 19136373 PMC2654976

[178] Sharron M Hoptay CE Wiles AA Garvin LM Geha M Benton AS. Platelets induce apoptosis during sepsis in a contact-dependent manner that is inhibited by GPIIb/IIIa blockade. PLoS ONE. (2012) 7:e41549. 10.1371/journal.pone.0041549 22844498 PMC3406039

[179] Mederle K Meurer M Castrop H Hocherl K. Inhibition of COX-1 attenuates the formation of thromboxane A2 and ameliorates the acute decrease in glomerular filtration rate in endotoxemic mice. Am J Physiol Renal Physiol. (2015) 309:F332–40. 10.1152/ajprenal.00567.2014 26017977

[180] Liverani E Rico MC Yaratha L Tsygankov AY Kilpatrick LE Kunapuli SP. LPS-induced systemic inflammation is more severe in P2Y12 null mice. J Leukoc Biol. (2014) 95:313–23. 10.1189/jlb.1012518 24142066 PMC4051260

[181] Liverani E Rico MC Tsygankov AY Kilpatrick LE Kunapuli SP. P2Y12 Receptor modulates sepsis-induced inflammation. Arterioscler Thromb Vasc Biol. (2016) 36:961–71. 10.1161/ATVBAHA.116.307401 27055904 PMC4850113

[182] Rahman M Gustafsson D Wang Y Thorlacius H Braun OO. Ticagrelor reduces neutrophil recruitment and lung damage in abdominal sepsis. Platelets. (2014) 25:257–63. 10.3109/09537104.2013.809520 23855479

[183] Totani L Dell'Elba G Martelli N Di Santo A Piccoli A Amore C. Prasugrel inhibits platelet-leukocyte interaction and reduces inflammatory markers in a model of endotoxic shock in the mouse. Thromb Haemost. (2012) 107:1130–40. 10.1160/TH11-12-0867 22436970

[184] Li X Li Y Shen K Li H Bai J. The protective effect of ticagrelor on renal function in a mouse model of sepsis-induced acute kidney injury. Platelets. (2018) 30:199–205. 10.1080/09537104.2017.1392499 29370574

[185] Claushuis TAM de Vos AF Roelofs J de Boer OJ van 't Veer C van der Poll T. Platelet-dense granules worsen pre-infection thrombocytopenia during gram-negative pneumonia-derived sepsis. J Innate Immun. (2018) 11:168–80. 10.1159/000494147 30557883 PMC6738263

[186] Seidel M Winning J Claus RA Bauer M Losche W. Beneficial effect of clopidogrel in a mouse model of polymicrobial sepsis. J Thromb Haemost. (2009) 7:1030–2. 10.1111/j.1538-7836.2009.03352.x 19548910

[187] Cornelius DC Baik CH Travis OK White DL Young CM Austin Pierce W. NLRP3 inflammasome activation in platelets in response to sepsis. Physiol Rep. (2019) 7:e14073. 10.14814/phy2.14073 31054188 PMC6499866

[188] Lopes Pires ME Antunes Naime AC Oliveira JGF Anhe GF Garraud O Cognasse F Signalling pathways involved in p47(phox) -dependent reactive oxygen species in platelets of endotoxemic rats. Basic Clin Pharmacol Toxicol. (2019) 124:394–403. 10.1111/bcpt.13148 30318767

[189] Liverani E Mondrinos MJ Sun S Kunapuli SP Kilpatrick LE. Role of Protein Kinase C-delta in regulating platelet activation and platelet-leukocyte interaction during sepsis. PLoS ONE. (2018) 13:e0195379. 10.1371/journal.pone.0195379 29617417 PMC5884571

[190] Steven S Dib M Roohani S Kashani F Munzel T Daiber A. Time response of oxidative/nitrosative stress and inflammation in LPS-induced endotoxaemia-A comparative study of mice and rats. Int J Mol Sci. (2017) 18:E2176. 10.3390/ijms18102176 29057830 PMC5666857

[191] Sellers RS Clifford CB Treuting PM Brayton C. Immunological variation between inbred laboratory mouse strains: points to consider in phenotyping genetically immunomodified mice. Vet Pathol. (2012) 49:32–43. 10.1177/0300985811429314 22135019

[192] Carreras E Velasco de Andres M Orta-Mascaro M Simoes IT Catala C Zaragoza O. Discordant susceptibility of inbred C57BL/6 versus outbred CD1 mice to experimental fungal sepsis. Cell Microbiol. (2018) 21:e12995. 10.1111/cmi.12995 30577088

[193] Wiedmeyer CE Ruben D Franklin C. Complete blood count, clinical chemistry, and serology profile by using a single tube of whole blood from mice. J Am Assoc Lab Anim Sci. (2007) 46:59–64. 17343355

[194] McKenzie SE Taylor SM Malladi P Yuhan H Cassel DL Chien P. The role of the human Fc receptor Fc gamma RIIA in the immune clearance of platelets: a transgenic mouse model. J Immunol. (1999) 162:4311–8. 10201963

[195] French SL Paramitha AC Moon MJ Dickins RA Hamilton JR Humanizing the protease-activated receptor (PAR) expression profile in mouse platelets by knocking PAR1 into the Par3 locus reveals PAR1 expression is not tolerated in mouse platelets. PLoS ONE. (2016) 11:e0165565 10.1371/journal.pone.0165565 27788223 PMC5082849

[196] Arachiche A de la Fuente M Nieman MT. Platelet specific promoters are insufficient to express protease activated receptor 1 (PAR1) transgene in mouse platelets. PLoS ONE. (2014) 9:e97724. 10.1371/journal.pone.0097724 24830314 PMC4022678

[197] Copeland S Warren HS Lowry SF Calvano SE Remick D Inflammation and the Host Response to Injury Investigators. Acute inflammatory response to endotoxin in mice and humans. Clin Diagn Lab Immunol. (2005) 12:60–7. 10.1128/CDLI.12.1.60-67.2005 15642986 PMC540200

[198] Warren HS Fitting C Hoff E Adib-Conquy M Beasley-Topliffe L Tesini B. Resilience to bacterial infection: difference between species could be due to proteins in serum. J Infect Dis. (2010) 201:223–32. 10.1086/649557 20001600 PMC2798011

